# A comparative study of electrochemical CO_2_ reduction on hydrothermally synthesized carbon nanosphere-supported Ni-, Cu-, and NiCu-hydroxide catalysts

**DOI:** 10.1039/d5cy01116g

**Published:** 2025-12-01

**Authors:** Yue Zhang, Qianqian Song, Jason M. J. J. Heinrichs, Marta Costa Figueiredo, Emiel J. M. Hensen

**Affiliations:** a Department of Chemical Engineering and Chemistry, Eindhoven University of Technology PO Box 513 Eindhoven 5600 MB The Netherlands e.j.m.hensen@tue.nl; b Eindhoven Institute of Renewable Energy Systems (EIRES), Eindhoven University of Technology PO Box 513 Eindhoven 5600 MB The Netherlands

## Abstract

The electrochemical reduction of CO_2_ (CO_2_RR) offers a promising route for sustainable fuel and chemical production. This study compares the CO_2_RR performance of hydrothermally synthesized carbon nanosphere-supported nickel hydroxide (Ni–C), copper hydroxide (Cu–C), and bimetallic nickel–copper hydroxide (NiCu–C) catalysts, investigating the influence of metal composition. Significant differences in product selectivity were observed: Cu–C primarily yielded C_2_ products, whereas Ni–C and NiCu–C generated mixtures of H_2_, CO, formate, and acetate, with minimal C_3_ products. Faradaic efficiencies (FEs) for C_3_ products (including propylene, propane, and *n*-propanol) were very low for Ni–C and NiCu–C (<0.3% combined). In comparison, Cu–C showed modest FEs (∼3–5%) primarily for *n*-propanol. X-ray photoelectron spectroscopy revealed partially oxidized nickel species (Ni^*δ*+^) in Ni–C and NiCu–C and predominantly Cu(i) species post-reaction, while scanning electron microscopy confirmed a distinct fibrous morphology for the Ni-containing catalysts. Control experiments with CO and acetate, and *in situ* Raman spectroscopy, suggest reaction pathways that differ from the typical Cu-catalyzed routes, potentially involving hydrogenated intermediates such as *CHO. This work provides a comparative analysis, highlighting how catalyst composition and associated electronic/structural properties influence the overall CO_2_RR activity and selectivity pathways in Ni, Cu, and NiCu hydroxide systems, rather than achieving significant C_3_ production.

## Abstract 2

Ni and Cu hydroxide catalysts are compared for CO_2_ electroreduction: Cu promotes C_2_ products *via* C–C coupling, while Ni^*δ*+^ sites enable a hydrogenation-driven route yielding trace C_3_ hydrocarbons.

## Graphical abstract

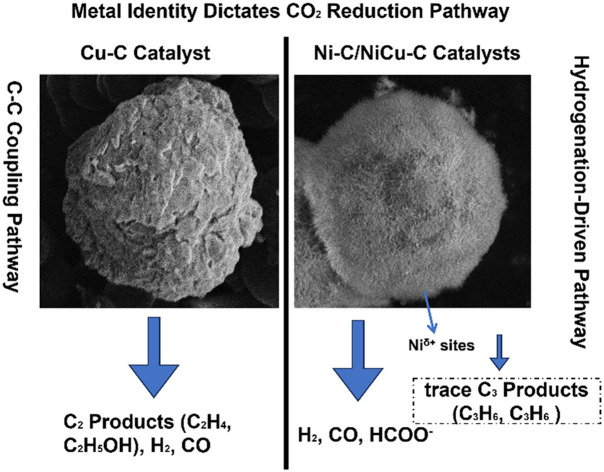

## Introduction

The escalating threat of climate change, driven by rising atmospheric CO_2_ levels, prompts the development of sustainable energy solutions. Electrochemical CO_2_ reduction (CO_2_RR) offers a potential approach to converting CO_2_ into valuable chemicals and fuels using renewable electricity, effectively mitigating greenhouse gas emissions and storing renewable energy in useful energy carriers and intermediate chemicals.^1–5^ CO_2_RR involves a series of proton-coupled electron transfer steps, in which CO_2_ molecules adsorbed on the surface of an electrocatalyst are reduced to various products, including carbon monoxide (CO), formic acid (HCOOH), methane (CH_4_), ethylene (C_2_H_4_), and longer-chain hydrocarbons. The overall reaction can be represented as:

1 CO_2_ + *n*H^+^ + *n*e^−^ → Products (CO, HCOOH, CH_4_, C_2_H_4_, C_3_H_8_, *etc.*)

The products formed depend on several factors, such as the applied potential, the electrolyte composition, and the nature of the electrocatalyst. The binding energies of the intermediates on the catalyst surface govern the selectivity towards different products. For example, strong binding of *CO generally favors the formation of hydrocarbons. However, too strong binding would hinder product desorption. In contrast, weak binding of *CO favors the production of CO.^6,7^

Among the products of CO_2_RR, those involving C–C bond formation (C_2+_ products) are highly desirable as energy carriers and chemical feedstock.^8^ Copper-based catalysts are unique in their ability to facilitate C–C coupling, particularly producing ethylene with promising selectivity, although they typically yield only small amounts of C_3+_ products.^9,10^ Achieving high selectivity for specific multi-carbon products requires careful catalyst design. Strategies explored include alloying,^11–14^ morphology control,^15–17^ and tandem approaches.^18,19^ aiming to develop highly efficient and selective catalysts, especially beyond C_2_ products, remains an active area of research.

Nickel-based materials have also emerged as electrocatalysts for CO_2_RR, often exhibiting different selectivity patterns compared to copper. Some studies suggest that specific nickel surface states, such as partially oxidized species (Ni^*δ*+^), might influence the formation pathways of multi-carbon products, albeit with modest yields.^20^ Understanding the intrinsic properties of nickel and copper, and how they interact in bimetallic systems, is crucial for tailoring catalyst performance. Combining copper's ability for C–C coupling with nickel's distinct surface chemistry presents an avenue for exploring novel catalytic behavior in bimetallic systems.

This work presents a comparative investigation of nickel hydroxide (Ni–C), copper hydroxide (Cu–C), and bimetallic nickel–copper hydroxide (NiCu–C) catalysts supported on carbon nanospheres for electrochemical CO_2_ reduction. These materials were synthesized *via* a hydrothermal approach, yielding distinct fibrous morphologies. We systematically compare their electrochemical activity and product selectivity across the C_1_–C_3_ range, correlating performance with material characteristics such as composition, surface state, and morphology. The potential roles of partially oxidized nickel sites (Ni^*δ*+^) and catalyst morphology in shaping overall reaction pathways and product distributions are explored. Furthermore, the influence of applied potential on product selectivity was investigated. Control experiments, including CO feeding, were used to probe reaction pathways and carbon sources, revealing differences compared to direct CO_2_ reduction. *In situ* Raman spectra provided further insights into potential surface intermediates and reaction mechanisms on these catalysts, exploring possibilities beyond conventional pathways.

## Experimental section

### Catalyst synthesis

Nickel hydroxide (Ni–C), copper hydroxide (Cu–C), and bimetallic nickel–copper hydroxide (NiCu–C) catalysts were synthesized on carbon nanosphere supports *via* a hydrothermal method. Carbon spheres were first prepared using a modified resorcinol-formaldehyde sol–gel method, followed by carbonization at 800 °C. For hydrothermal synthesis, aqueous solutions of metal nitrates (Ni(NO_3_)_2_·6H_2_O and/or Cu(NO_3_)_2_·3H_2_O) were mixed with urea and the carbon spheres, then heated at 120 °C for 6 h. For comparison, a NiO–C catalyst was prepared by annealing the Ni–C powder at 400 °C in air. Commercial NiO powder was used as received. Complete synthetic protocols are provided in the SI.

### Materials characterization

Catalyst morphology and composition were examined using scanning electron microscopy (SEM) with energy-dispersive X-ray spectroscopy (EDX). Crystalline phases were identified by X-ray diffraction (XRD). Surface elemental composition and chemical states were analyzed with X-ray photoelectron spectroscopy (XPS) before and after electrolysis. *In situ* Raman spectroscopy was employed to probe surface species during CO_2_ reduction. Detailed instrument parameters and sample preparation procedures for all characterization techniques are available in the SI.

### Electrochemical measurements

Electrochemical CO_2_ reduction was performed in a gas-tight, two-compartment H-cell separated by a Nafion 117 membrane. A three-electrode configuration was used, with the catalyst drop-cast onto a glassy carbon electrode as the working electrode, a Pt foil counter electrode, and an Ag/AgCl reference electrode. All potentials were corrected for 80% of the uncompensated resistance (iR) and are reported *versus* the reversible hydrogen electrode (RHE).

Chronoamperometry experiments were conducted at potentials from −0.6 to −1.1 V *vs.* RHE in CO_2_-saturated 0.1 M KHCO_3_ electrolyte. Gaseous products were quantified by online gas chromatography (GC) and liquid products by 1H nuclear magnetic resonance (NMR) spectroscopy. The electrochemically active surface area (ECSA) was estimated from Pb underpotential deposition (Pb-UPD) measurements. Further details on the electrochemical setup, product analysis protocols, control experiments, and ECSA calculations are provided in the SI.

## Results and discussion

### Catalyst synthesis and characterization

Nickel–copper bimetallic hydroxide anchored on carbon spheres (NiCu–C), along with the corresponding nickel (Ni–C) and copper (Cu–C) monometallic reference catalysts, were synthesized through a hydrothermal approach, using methods adapted from the literature.^21^ The carbon spheres were prepared from resorcinol and formaldehyde through a sol–gel method followed by carbonization.^22^ These porous spheres served as a support for subsequent hydrothermal synthesis, where nickel nitrate and copper nitrate were used as metal precursors. A Ni/Cu molar ratio of 2 was chosen, following the established synthesis protocol developed by our co-author,^21^ and urea served as a precipitating agent to form the hydroxides (see SI for full experimental details). The catalysts were then deposited onto glassy carbon electrodes with a mass loading of ∼0.53 mg cm^−2^.

**Fig. 1:**
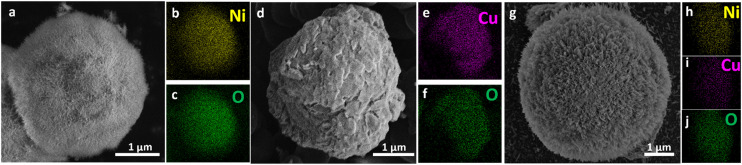
SEM and EDX mapping images of a–c) Ni–C; d–f) Cu–C; and g–j) NiCu–C.

SEM and EDX mapping revealed distinct morphological and compositional characteristics (Fig. 1 and S3). SEM imaging shows that the Ni–C catalyst is made of spherical particles with a highly textured, fibrous surface, indicative of a porous architecture (Fig. 1a). This fibrous morphology is expected to provide a high surface area and facilitate mass transport during the electrochemical reaction. EDX mapping confirms the presence of nickel and oxygen (Fig. 1b and c). The Cu–C catalyst displays irregular particles with a rough, granular surface (Fig. 1d). The EDX maps confirm the presence of copper and oxygen (Fig. 1e and f). Finally, the bimetallic NiCu–C catalyst consists of densely fibrous spherical particles (Fig. 1g), morphologically similar to Ni–C, suggesting the porous structure is retained upon copper incorporation. The EDX maps in Fig. 1h–j reveal a uniform distribution of nickel, copper, and oxygen at the micrometer scale. The carbon support morphology consists of smooth spheres (Fig. S4). EDX analysis of the NiCu–C catalyst indicated a Ni/Cu atomic ratio of 3.65, which deviates from the target ratio of 2. This difference could be attributed to variations in the incorporation of Ni and Cu during hydrothermal synthesis. The metal loadings on the catalysts are 74.4 μg Ni per cm^2^ for Ni–C, 56.9 μg Cu per cm^2^ for Cu–C and 52.9 μg Ni per cm^2^ and 15.7 μg Cu per cm^2^ for NiCu–C catalyst (see SI). The surface Ni/Cu ratio of 4 determined by X-ray photoelectron spectroscopy (XPS) is in reasonable agreement with the EDX analysis. The slight difference between the surface and bulk Ni/Cu ratios suggests a possible surface enrichment of Ni.

**Fig. 2:**
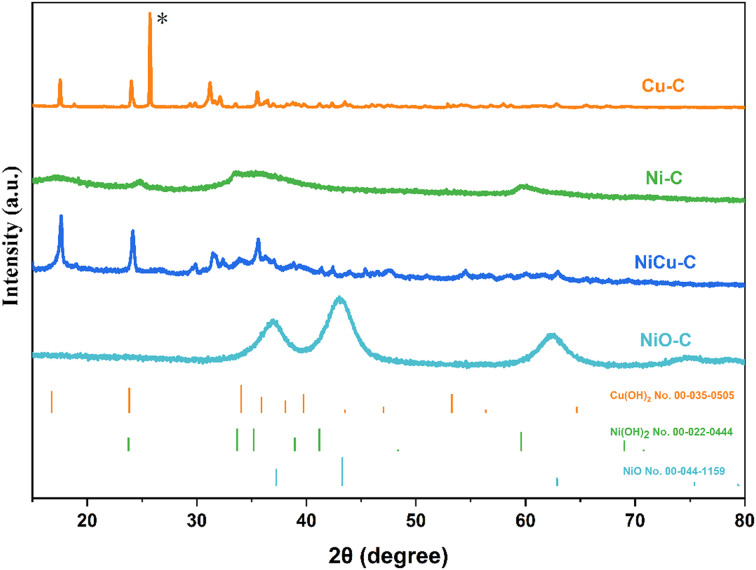
XRD patterns of Cu–C, Ni–C, NiCu–C and NiO–C, the reference samples are listed at the bottom.

To further investigate the crystallographic structures of the crystalline phases, XRD analysis was performed (Fig. 2). The XRD pattern of the Cu–C catalyst (Fig. 2) exhibits peaks at 2*θ* = 16.7°, 23.8°, and 34.1°, which can be indexed to the (020), (021), and (002) planes of orthorhombic Cu(OH)_2_ (JCPDS No. 00-035-0505), respectively. A intense peak, which can be attributed to the (002) reflection of the graphitic carbon sphere support is observed at around 26.5°,^23^ is particularly apparent in the Cu–C sample, while less defined in the other samples. The XRD pattern of the Ni–C sample (Fig. 2) displays broad diffraction peaks at around 2*θ* = 33.6°, 35.2° and 59.6°, which are consistent with the (110), (111) and (300) reflections of hexagonal Ni(OH)_2_ (JCPDS No. 00-022-0444). The broadness of these peaks suggests the nanocrystalline nature of the Ni(OH)_2_. While the Scherrer equation is most accurately applied to ideal spherical crystallites, it can be used to estimate an apparent crystallite size for the non-spherical, fibrous nanostructures observed in our Ni(OH)_2_ sample.^24^ Using the (110) peak, an apparent crystallite size of approximately 2.8 nm was calculated. This value is understood to primarily reflect the average dimension perpendicular to the (110) diffracting planes, corresponding to the average diameter or width of the Ni(OH)_2_ fibers. It is important to note that this estimate does not directly reflect the fibers' length, which is likely longer. The XRD pattern of the bimetallic NiCu–C catalyst contains peaks characteristic of both Cu(OH)_2_ and Ni(OH)_2_. It is worth noting that the peaks associated with Ni(OH)_2_ are broader and less intense in the NiCu–C sample compared to the Ni–C sample. This could be attributed to a smaller Ni(OH)_2_ crystallite size in the bimetallic catalyst or to a lower degree of crystallinity. To further investigate the effect of thermal treatment on the nickel-based catalyst, the Ni–C sample was annealed at 400 °C in air to form NiO–C. The XRD pattern of NiO–C (Fig. 2) reveals distinct peaks at 2*θ* = 37.3°, 43.3°, and 62.9°, corresponding to the (101), (012), and (110) planes of cubic NiO (JCPDS No. 00-044-1159), respectively. These features confirm the successful conversion of Ni(OH)_2_ to NiO upon annealing. The broad peaks observed in the NiO–C sample point to the formation of very small NiO crystallites.

To further elucidate the catalyst nanostructure, transmission electron microscopy (TEM) analysis was performed (see Fig. S24 and S25 in the SI). Bright-field TEM (BF-TEM) images of the monometallic Ni–C catalyst confirmed its nanocrystalline nature, revealing finely dispersed crystalline domains consistent with the ∼2.8 nm crystallite size determined by XRD. For the bimetallic NiCu–C catalyst, high-angle annular dark-field scanning TEM (HAADF-STEM) coupled with energy-dispersive X-ray (EDX) mapping was performed (Fig. S26). The HAADF-STEM images and corresponding EDX maps show uniform nanoscale mixing of Ni and Cu, with no evidence of phase separation or large domains of either metal.

### Surface states and species after reaction

**Fig. 3:**
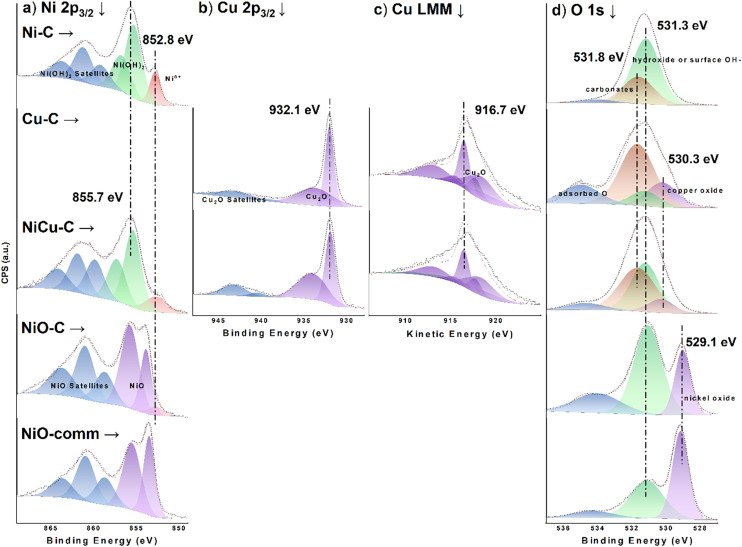
XPS spectra of (a) Ni 2p_3/2_, (b) Cu 2p_3/2_, (c) Cu LMM, and (d) O 1s regions for Ni–C, Cu–C, NiCu–C, NiO–C, and NiO-comm samples after CO_2_RR. Blank spaces in (b) and (c) indicate the absence of a detectable Cu signal.

XPS was employed to investigate the surface speciation and oxidation states of Cu and Ni on the mono- and bimetallic catalysts after CO_2_RR. The Cu 2p_3/2_ spectra of as-prepared Cu–C and NiCu–C samples (Fig. S5b) showed prominent peaks at 934.5 eV and satellite features, along with a Cu LMM feature at 916.3 eV (Fig. S5c), all consistent with literature values for Cu(OH)_2_.^25,26^ Following the CO_2_RR reaction, the Cu 2p_3/2_ spectra of Cu–C and NiCu–C (Fig. 3b) showed a prominent peak at around 932.1 eV with reduced satellite peaks, suggesting a majority of Cu^+^ and/or Cu^0^ species. Confirmation of the Cu^+^ state is provided by the Cu LMM Auger spectra (Fig. 3c), where the prominent peak at approximately 916.7 eV is characteristic of Cu_2_O.^25,26^ This indicates a transformation during the reaction, where the initial Cu(OH)_2_ was likely reduced to metallic Cu and subsequently re-oxidized to Cu_2_O upon exposure to air. This is consistent with previous studies showing that metallic Cu readily oxidizes to Cu_2_O in air.^27–29^ The O 1s spectra further support the Cu–C bond transition after CO_2_RR. Specifically, the O 1s spectra for Cu–C after CO_2_RR (Fig. 3d) show a decrease in the intensity of the peak associated with hydroxide species (around 531.0–532.5 eV) and an increase in the intensity of the peak related to oxide species (around 529.0–530.5 eV), compared to the spectra of the as-prepared sample (Fig. S5d).^30–32^ While a similar shift might be expected for NiCu–C, it is likely obscured by the dominant presence of the stable Ni(OH)_2_ phase, which also contributes to the hydroxide peak. Furthermore, the significantly lower Cu content in NiCu–C compared to Cu–C likely results in a less pronounced change in the O 1s spectrum. However, it is important to acknowledge that Ni(OH)_2_ can form surface carbonates upon exposure to air.^33^ The O 1s signal of carbonates (typically around 531.5–532 eV) overlaps with that of hydroxides, complicating the analysis. Therefore, the peaks around 531–532 eV in Ni–C, Cu–C, and NiCu–C spectra may represent a combination of hydroxide and carbonate species.

In contrast to the observed transformation of Cu species, the Ni 2p spectra of Ni–C and NiCu–C (Fig. 3a and S4a) showed minimal changes after the reaction, suggesting that the Ni species remained primarily in the Ni(OH)_2_ phase. The main Ni 2p_3/2_ peaks for Ni–C and NiCu–C were observed at 855.7 eV, respectively (Fig. 3a). This binding energy value, together with the strong satellite peaks at higher binding energies, is consistent with those reported in the literature for Ni(OH)_2_,^31,34^ suggesting that the Ni(OH)_2_ phase remained largely stable during CO_2_RR. Previous studies further support this stability.^35^ Additionally, our *in situ* Raman spectroscopy results (see details later and Fig. 8) confirm the presence of Ni(OH)_2_ during the reaction. In addition to the main Ni^2+^ peaks, a minor component was observed at a slightly lower binding energy of approximately 852.8 eV in the Ni 2p_3/2_ region for both Ni–C and NiCu–C, respectively (Fig. 3a). While this binding energy region could potentially be attributed to metallic Ni, the dominant presence of Ni(OH)_2_ features in the spectra, combined with the absence of a clear metallic Ni peak around 852.3–852.6 eV,^31,36^ suggests that this component more likely corresponds to a slightly electron-deficient Ni species (Ni^*δ*+^, where 0 < *δ* < 2) within the hydroxide structure or at the interface between the Ni(OH)_2_ and the carbon support. It is worth noting that the pre-treatment at −0.5 V *vs.* RHE was primarily intended to remove surface oxides formed during air exposure and it is generally not considered to be a sufficiently reducing potential to cause significant reduction of Ni(OH)_2_ to metallic Ni under these conditions.^35^ The assignment of the peak at 852.8 eV to Ni^*δ*+^ is consistent with previous studies that have reported similar shifts for Ni species in close interaction with conductive supports or other element species.^37–40^ The exact nature of this Ni^*δ*+^ species and its potential role in catalysis require further investigation. However, we do not find evidence for significant amounts of metallic Ni in our samples under these conditions. The distinctive production of propylene and propane on both Ni–C and NiCu–C might thus be related to the presence of the Ni^*δ*+^ species within the Ni(OH)_2_ matrix. However, the exact role of this species remains to be investigated.^41,42^

XPS analysis of NiO–C after CO_2_RR (Fig. 3a) also confirmed the presence of a Ni^*δ*+^ species, similar to the post-reaction Ni–C case. Notably, the Ni 2p_3/2_ region of NiO–C revealed a significantly higher Ni^2+^/Ni^*δ*+^ ratio of approximately 19.0, compared to the ratios observed for Ni–C (6.1) and NiCu–C (10.1) after CO_2_RR. (Table S8). This higher ratio in NiO–C is likely due to the thermal treatment during its synthesis, which favors the formation of stoichiometric NiO with fewer defects or interstitial sites that could host Ni^*δ*+^. In contrast, the XPS analysis of the commercial NiO (Fig. 3a) indicated that the surface consisted primarily of NiO. Differences in Ni oxidation states and Ni^2+^/Ni^*δ*+^ ratios between the synthesized catalysts and commercial NiO could contribute to their distinct catalytic performance, as discussed in the following sections.

These characterization results provide a comprehensive understanding of morphology, composition, and surface properties of the synthesized catalysts. The fibrous morphology of Ni–C and NiCu–C, the presence of both Ni and Cu species in NiCu–C, and the identification of Cu_2_O and Ni^*δ*+^ species after CO_2_RR are particularly noteworthy. Further investigation will focus on the correlation between these characteristics and their effects on catalytic performance.

### Electrocatalytic performance

Electrochemical CO_2_ reduction experiments were conducted in a typical H-cell setup.^43^ Before the reaction experiments, the electrocatalysts were conditioned at −0.5 V *vs.* RHE in a 1 M KHCO_3_ solution for 1 h to remove surface oxides and ensure a consistent starting surface. Gaseous products were quantified using online gas chromatography, while liquid products were analyzed using ^1^H nuclear magnetic resonance (NMR) spectroscopy of the electrolyte after the experiment.

**Fig. 4:**
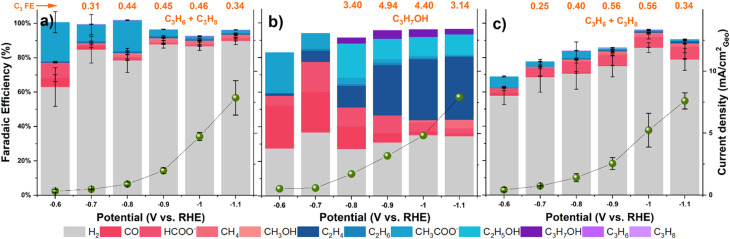
Faradaic efficiency of products on a) Ni–C, b) Cu–C and c) NiCu–C catalysts during CO_2_ reduction in 0.1 M KHCO_3_ solution. C_3_ products FEs are labelled for easy comparison. Error bars for (a) and (c) represent the standard deviation from independent replicate measurements; values in panel (b) are from a single benchmark experiment.

Fig. 4 presents the faradaic efficiencies (FEs) and current densities across a potential range of −0.6 to −1.1 V *vs.* RHE. Significant differences in product distribution were observed between the catalysts. Ni–C mainly produced H_2_ along with CO, formate, acetate, and very small amounts of C_3_ hydrocarbons (propylene + propane combined FE ≤ 0.46% at −1.0 V *vs.* RHE; Table S3). Cu–C, consistent with literature, mainly yielded C_2_ products (ethylene, acetate, ethanol) and CO, with H_2_ as a major co-product. Notably, Cu–C produced primarily *n*-propanol as its C_3_ product, reaching modest FEs of 3–5% at investigated potentials, while producing only trace propylene (≤0.1%) and no detectable propane (Table S4). The bimetallic NiCu–C catalyst exhibited a product spectrum somewhat similar to Ni–C regarding C_1_/C_2_ products, favoring HCOO^−^ production over CH_3_COO^−^. It produced C_3_ hydrocarbons (propylene + propane) with FEs comparable to Ni–C, reaching a maximum combined FE of 0.56% at −0.9 V *vs.* RHE (Table S5). Crucially, FEs for C_3_ hydrocarbons on both Ni–C and NiCu–C were consistently below 0.6%, representing minor product channels.

Comparing selectivity trends (Fig. 4), Cu–C showed increasing C_2_H_4_ FE at more negative potential, coupled with decreasing CO FE, typical for Cu-catalyzed CO coupling.^44,45^ In contrast, NiCu–C and Ni–C showed less pronounced changes in CO FE. An interesting trend was observed for the C_3_H_6_/C_3_H_8_ ratio on the Ni-containing catalysts, which increased at more negative potentials, particularly for NiCu–C, possibly indicating potential-dependent hydrogenation pathways.

The stark difference in selectivity for the NiCu–C catalyst, which lacks the formation of C_2_H_4_ and *n*-propanol as observed for Cu–C, is critical. We attribute the suppression of C–C coupling to an ensemble effect: the small number of Cu atoms, well dispersed in the initial dominant Ni(OH)_2_ matrix, does not form Cu surface ensembles amenable to C–C coupling reactions. Furthermore, the suppression of acetate (a C_2_ product dominant on Ni–C; see Fig. 4a) in favor of formate on the NiCu–C catalyst suggests that these dispersed Cu atoms alter the electronic properties of neighboring Ni sites, thereby inhibiting acetate formation.

The overall geometric current densities were comparable across the three catalysts under these conditions (Fig. 4), suggesting that the observed differences in product selectivity likely stem from intrinsic kinetic differences rather than significant variations in accessible surface area. Electrochemically active surface area (ECSA) estimation *via* double-layer capacitance yielded potentially underestimated roughness factor (RF) values (RF ∼0.43 for NiCu–C) due to the pseudo-capacitive contributions of the carbon support and metal hydroxide phases. Underpotential deposition of Pb (Pb-UPD) measurements indicated a much larger ECSA (RF ∼26.2 for NiCu–C). While Pb-UPD on Ni-containing materials has limitations due to oxide formation and alloying, the higher value aligns better with the fibrous morphology observed in the SEM images and the small apparent crystallite size (∼2.8 nm) from XRD, suggesting a higher intrinsic surface area for these materials.

### Comparison with NiO catalysts and role of morphology

**Fig. 5:**
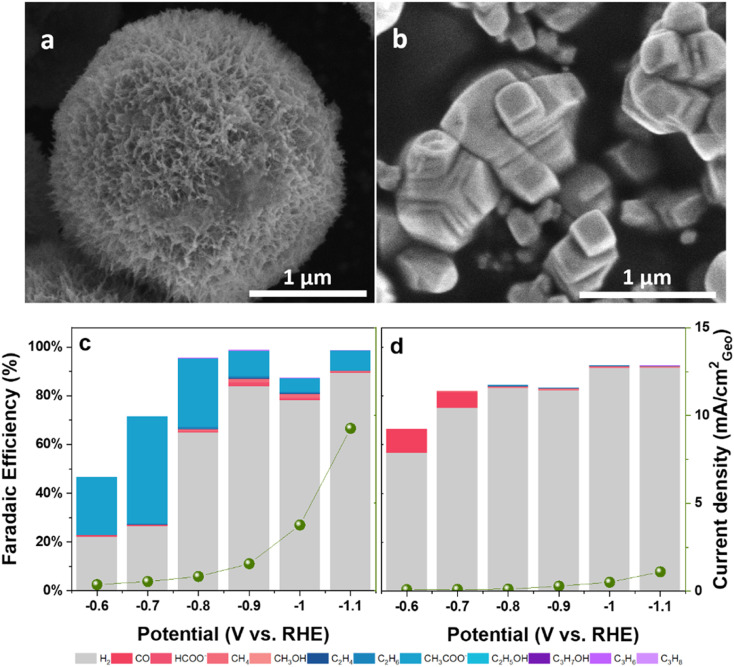
a and b) SEM images; c and d) faradaic efficiency of products on NiO–C (left) and commercial NiO (right) catalysts during CO_2_ reduction in 0.1 M KHCO_3_ solution.

To further explore factors influencing selectivity, the performance of NiO–C (annealed Ni–C at 400 °C in air for 4 h) and commercial NiO was evaluated. NiO–C retained the fibrous morphology of its precursor (Fig. 5a), while commercial NiO exhibited a densely packed, non-porous morphology, typical of bulk materials (Fig. 5b). NiO–C exhibited CO_2_RR performance similar to Ni–C, producing C_3_ hydrocarbons with a maximum combined FE of 0.46% at −0.9 V *vs.* RHE (compared to 0.45% for Ni–C at same potential; Tables S3 and S6). In contrast, commercial NiO showed significantly lower activity towards C_3_ hydrocarbons (combined FE ∼0.13% only at −1.1 V *vs.* RHE; Table S7) and different overall selectivity (Fig. 5c and d). This comparison suggests that the presence of Ni^*δ*+^ species (identified by XPS in both Ni–C and NiO–C, but largely absent in commercial NiO) correlates with the ability to produce even trace amounts of C_3_ hydrocarbons. However, the very low FEs achieved make it difficult to definitively assign a crucial role to Ni^*δ*+^ solely for C_3_ production, as these surface states likely influence other reaction pathways as well. The fibrous morphology shared by Ni–C, NiCu–C, and NiO–C, contrasting with the dense commercial NiO, likely also plays a role. The high surface area and porosity could enhance mass transport and potentially influence local pH *via* restricted diffusion, affecting overall activity and product distribution, although its specific impact on the minor C_3_ pathway is difficult to isolate. The fibrous structure demonstrated good stability over 12 h (Fig. S10 and S11), maintaining C_3_ FE with only a slight current density decrease.

### Role of CO and acetate as potential intermediates for multi-carbon products

Previous studies suggest that adsorbed *CO is a key intermediate for C_2+_ products in CO_2_RR, particularly on Cu-based catalysts where it undergoes coupling, similar to CO electroreduction (CORR) processes.^5,44,46^ To understand the behavior and potential role of *CO on our Ni-based catalysts, we examined its FE as a function of applied potential. In our experiments, a slight decrease in the FE of CO on both Ni–C and NiCu–C was observed as the potential became more negative. However, this decrease was less pronounced than that observed for Cu–C. Additionally, a consistent decrease in CH_3_COO^−^ FEs was noted across the potential range for both Ni–C and NiCu–C catalysts (Fig. 4 and Tables S3 and S5).

**Fig. 6:**
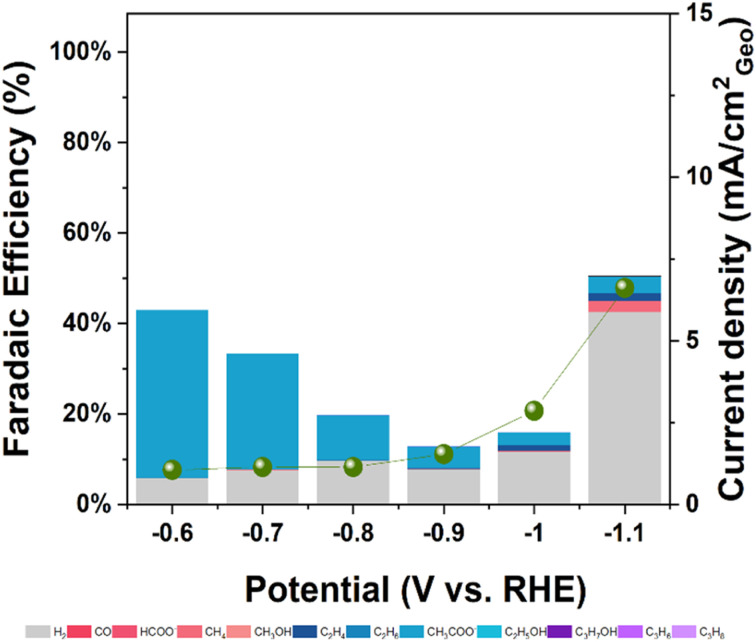
Faradaic efficiency of products on NiCu–C catalyst during CO reduction in 0.1 M KHCO_3_ solution.

Control experiments were conducted to probe reaction intermediates using CO instead of CO_2_ as the reactant with the NiCu–C catalyst (0.1 M KHCO_3_ solution). Under these conditions, two notable observations were made compared to when CO_2_ was the feedstock: total faradaic efficiencies were below unity, and hydrogen production was significantly suppressed (Fig. 6 and Table S9). Both of these effects may be attributed to strong CO binding on nickel sites, limiting the availability of active sites, thereby reducing total FE, and simultaneously hinder reactants access for CO reduction and hydrogen evolution.^47,48^ Notably, the FEs for propylene and propane with CO reactant were significantly lower (≤0.1%) than those observed with CO_2_ reactant. This suggests CO is likely not the sole or primary C_1_ intermediate leading to the observed C_3_ hydrocarbons when starting from CO_2_.

**Fig. 7:**
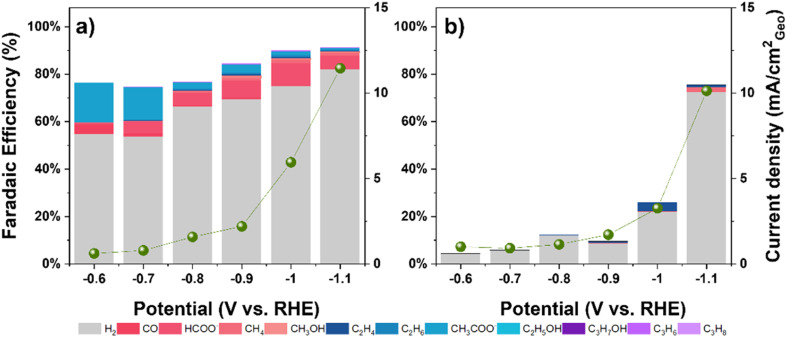
Faradaic efficiency of products on NiCu–C catalyst during a) CO_2_ reduction and b) CO reduction in 0.1 M KHCO_3_ + 0.05 M KOAc solution.

Given the consistent decrease in acetate FEs as the applied potentials became more negative in both CO_2_RR and CORR, experiments substituting acetate (KOAc electrolyte) for bicarbonate under various feeding conditions (He, CO, and CO_2_, with and without applied potentials) were then carried out (Fig. S6 and Tables S10–S12). In a He atmosphere, only H_2_ was produced (Fig. S6), highlighting that the C_3_ products do not derive from other carbon-containing sources. Using CO in the feed in the KOAc electrolyte, no improvement in carbon-containing products was observed compared to using CO_2_ in the feed (Fig. S6b), similar with the results obtained in 0.1 M KHCO_3_. Conversely, the use of CO_2_ in the KOAc electrolyte yielded similar FEs for propylene and propane, along with other products, as observed in the KHCO_3_ electrolyte (Fig. S6c). These results showed no enhancement of C_3_ products from either CO or CO_2_ feed compared to KHCO_3_, indicating acetate is not a primary intermediate for C_3_ formation under these conditions. To further investigate this and address potential pH effects, a mixed electrolyte containing 0.05 M KOAc and 0.1 M KHCO_3_ was applied. This approach was chosen to mitigate the lower pH of the pure 0.1 M KOAc electrolyte (pH 5.8) relative to 0.1 M KHCO_3_ (see pH values in Table S13), as local pH can influence product distribution.^49–51^ Experiments using this mixed electrolyte yielded similar product distributions to those obtained with KHCO_3_ alone (Fig. 7 and Tables S14 and S15). This outcome reinforces the conclusion that acetate is not a primary intermediate for C_3_ formation under these conditions.

Recent studies suggested that nickel-based electrocatalysts for CO_2_RR may operate *via* a mechanism similar to Fischer–Tropsch synthesis (FTS).^20,52^ We therefore examined whether our catalysts follow an Anderson–Schulz–Flory (ASF) distribution, characteristic of the classical Fischer–Tropsch process and allowing determination of the chain-growth probability, α. An ASF analysis including C_1_, C_2_, and C_3_ products was performed, similar to an approach reported in recent literature (Fig. S8).^52^ Our analysis revealed that the conditions yielding the highest FEs for C_3_ gaseous products also corresponded to a linear ASF plot. Specifically, the chain-growth probabilities α (based on C_1_–C_3_ products) were calculated to be 0.30 and 0.34 for Ni–C and NiCu–C electrocatalysts at −1.0 V *vs.* RHE, respectively. At other potentials, deviations from the ideal ASF distribution are observed. The formation of formate and acetate likely occurs through alternative pathways, as they are not typically involved in the chain-growth mechanism of FTS. Although our results point toward a Fischer–Tropsch-like mechanism on the Ni–C and NiCu–C catalysts, we present this only as a tentative pathway. A detailed investigation of this hypothesis lies beyond the scope of the present study.

### Raman spectroscopy

**Fig. 8:**
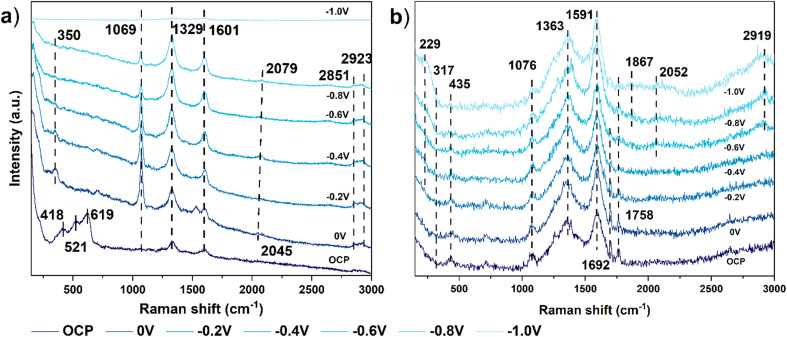
*In situ* Raman spectra of a) Cu–C and b) Ni–C in CO_2_ saturated 0.1 M KHCO_3_ from OCP to −1.0 V *vs.* RHE.

*In situ* Raman spectroscopy was employed to investigate surface intermediates during CO_2_RR. Given the complexity of analyzing multicomponent samples with this technique, the analyses focused on the Cu–C and Ni–C catalysts to avoid potential interferences from the bimetallic catalyst. Raman spectra were recorded at open circuit potential (OCP; Cu–C: −0.02 V and Ni–C: 0.18 V *vs.* Ag/AgCl) and applied potentials ranging from 0 to −1.0 V *vs.* RHE with 200 mV intervals (Fig. 8). To minimize the influence of surface layers that could form during air exposure, electrodes were pre-treated at −0.6 V *vs.* RHE for 15 min before recording spectra at each potential.^53,54^

Raman spectroscopy of the Cu–C catalyst revealed characteristic peaks of surface Cu_2_O at OCP (418, 521, and 619 cm^−1^), which disappeared upon bias application (Fig. 8a), suggesting the reduction of surface Cu_2_O to metallic Cu under the applied potentials. This rapid formation of Cu_2_O upon air exposure aligns with previous studies.^28,55^ Strong Raman peaks at 1329 cm^−1^ and 1601 cm^−1^ corresponded to the D and G bands, respectively, characteristic of disordered carbon materials.^56,57^ with the peak at 1069 cm^−1^ assigned to symmetric vibrations of the carbonate ion CO_3_^2−^.^58–60^ Upon bias application, a new peak emerged at 350 cm^−1^, indicative of the Cu–CO stretching mode.^58,59^ Additionally, the region related to CO adsorption and CH_*x*_ species (1800–3000 cm^−1^) revealed the presence of linear-bonded CO (2045 cm^−1^) at the initial potential,^61,62^ alongside symmetric –CH_2_ (*ν*_s_CH_2_) and –CH_3_ (*ν*_s_CH_3_) stretching vibrations (2851 cm^−1^ and 2923 cm^−1^, respectively),^63^ suggesting the conversion of adsorbed CO on Cu–C. At more negative potentials, the linear-bonded CO peak shifted to 2079 cm^−1^, potentially indicating high CO coverage, which is known to favor C–C coupling and subsequent ethylene production.^64–66^ These findings are consistent with previous Raman studies of Cu-based catalysts during CO_2_RR.^62,63^

The Raman spectrum of the Ni–C catalyst exhibited three characteristic peaks corresponding to the carbon support and carbonate, similar to those observed on Cu–C (1363, 1591, and 1076 cm^−1^, respectively) (Fig. 8b). Additionally, two peaks at 317 and 435 cm^−1^, attributed to Ni(OH)_2_, persisted within the target potential range, suggesting its stability during CO_2_RR.^67–69^ This is consistent with our XPS results, which showed that the Ni species remained primarily in the Ni(OH)_2_ phase after CO_2_RR. The peak at 1692 cm^−1^, attributable to C=O asymmetric stretching associated with *COOH, diminished with increasing bias, correlating with its FE performance.^70,71^ Concurrently, weak and broad features in the region of the Ni–CO stretching mode (229 cm^−1^), bridge-bonded (1867 cm^−1^), and linear-bonded CO (2052 cm^−1^) at −0.6 V *vs.* RHE as the 1692 cm^−1^ peak weakened, suggesting the adsorption of CO on the Ni surface.^72^ A significant –CH_3_ (*ν*_s_CH_3_) stretching peak at 2919 cm^−1^ also became prominent at −0.6 V *vs.* RHE, aligning with the onset of propylene and propane at −0.7 V *vs.* RHE. Unlike Cu–C, Ni–C exhibited a feature at 1758 cm^−1^, which we tentatively assigned to *CHO.^73,74^ Conclusive identification will require isotope-labeling studies. This peak may indicate the presence of hydrogenated species, and *CHO is considered a key intermediate in the hydrogenation of *CO to form CH_4_.^75,76^

The CO adsorption region exhibited distinct behaviors of each catalyst. On Cu–C, linear-bonded CO was observed at all investigated potentials, whereas on Ni–C, broad linear-bonded CO (2052 cm^−1^) emerged at −0.6 V *vs.* RHE and bridge-bonded CO (1867 cm^−1^) at −0.8 V *vs.* RHE. Based on the relative intensities of the CO stretching modes, this indicates a comparatively lower *CO coverage on Ni–C than on Cu–C. These observations suggest possible differences in intermediate pathways: the presence of *CHO and lower *CO coverage on Ni–C might relate to its different selectivity profile (*e.g.*, higher H_2_, lower C_2+_) compared to Cu–C, favoring hydrogenation pathways over C–C coupling, which is typical on Cu.^44,75,77^ The Raman data did not support acetate as a key surface intermediate, aligning with electrochemical controls.

## Conclusions

This work presented a comparative investigation of the electrochemical CO_2_ reduction performance of hydrothermally synthesized nickel hydroxide (Ni–C), copper hydroxide (Cu–C), and bimetallic nickel–copper hydroxide (NiCu–C) catalysts supported on carbon spheres. The study revealed distinct catalytic behavior and product distributions dictated by the metal composition. Copper-based catalysts (Cu–C) primarily favored C_2_ products (ethylene, ethanol, acetate) alongside CO and H_2_, consistent with known Cu-catalyzed CO_2_RR pathways. Cu–C also produced modest amounts (∼3–5% FE) of *n*-propanol. In contrast, nickel-based catalysts (Ni–C and NiCu–C) exhibited different selectivity profiles, predominantly yielding H_2_, CO, formate, and acetate, along with trace amounts of C_3_ hydrocarbons (propylene and propane combined FE < 0.6%). A key aspect highlighted by this comparative analysis is the challenge associated with minor product channels. It is important to note that accurate quantification of products at the very low faradaic efficiencies observed for C_3_ compounds on Ni–C and NiCu–C presents significant analytical challenges and inherent uncertainties. Characterization revealed differences in material properties correlating with the observed electrochemical behavior. The distinct fibrous morphology of Ni–C and NiCu–C, in contrast to the granular Cu–C morphology, likely influences mass transport and surface area. Post-reaction surface analysis indicated the stability of Ni(OH)_2_ (with associated Ni^*δ*+^ species) on Ni-based catalysts, whereas Cu(OH)_2_ was reduced, resulting in predominant Cu(i) species after air exposure. While the presence of Ni^*δ*+^ species correlated with the detection of trace C_3_ hydrocarbons, the low yields preclude assigning a definitive promotional role based solely on this study. Mechanistic probes, including control experiments and *in situ* Raman spectroscopy, suggested differing reaction pathways on the catalyst surfaces. Evidence indicated that CO was not the sole C_1_ intermediate on the Ni-based catalysts for the observed product distribution, suggesting the potential involvement of hydrogenated species such as *CHO, unlike in typical Cu-catalyzed routes. Thus, this comparative study highlights the strong influence of metal composition (Ni *vs.* Cu *vs.* NiCu) on CO_2_RR pathways and product selectivity using hydroxide-based catalysts. While Ni-containing catalysts enabled the formation of C_3_ hydrocarbons not readily observed on Cu–C, the yields were minimal under the conditions tested. Future research could focus on further understanding the fundamental mechanistic differences between Ni and Cu surfaces, the precise role of surface states such as Ni^*δ*+^, and leveraging morphology control to potentially improve selectivity towards desired multi-carbon products, while acknowledging the significant challenge of achieving high C_3_ yields with these systems.

## Author contributions

Yue Zhang: conceptualization, investigation, data analysis, writing – original draft and editing. Qianqian Song: investigation and data analysis. Jason M. J. J. Heinrichs: TEM measurements and analysis. Marta Costa Figueiredo: conceptualisation, supervision, writing – original and revised drafts, and editing. Emiel J. M. Hensen: conceptualization, supervision, writing – original and revised drafts and editing, funding acquisition.

## Conflicts of interest

There are no conflicts to declare.

## Supplementary Material

CY-016-D5CY01116G-s001

## Data Availability

The authors confirm that the data supporting the findings of this study are available within the article and its supplementary information (SI). Supplementary information is available. See DOI: https://doi.org/10.1039/d5cy01116g.
