# Supplementary material for: A comparative study of electrochemical CO2 reduction on hydrothermally synthesized carbon nanosphere-supported Ni-, Cu-, and NiCu-hydroxide catalysts
Source: Catal Sci Technol. 2025 Dec 1;16(1):327–37. doi: 10.1039/d5cy01116g (PMC12667018; doi:10.1039/d5cy01116g)
Supplement: CY-016-D5CY01116G-s001 [file CY-016-D5CY01116G-s001.pdf]

Supplementary Information (SI) for Catalysis Science & Technology.
This journal is © The Royal Society of Chemistry 2025

Supplementary Information

A Comparative Study of Electrochemical CO_2 Reduction on Hydrothermally Synthesized Carbon Nanosphere-Supported Ni-, Cu-, and NiCu-hydroxide Catalysts

Yue Zhang,[a] Qianqian Song,[a] Jason M.J.J. Heinrichs,[a] Marta Costa Figueiredo,[a,b] and Emiel J. M. Hensen[a]

[a] Department of Chemical Engineering and Chemistry, Eindhoven University of Technology, PO Box 513, Eindhoven 5600 MB, the Netherlands, E-mail: e.j.m.hensen@tue.nl

[b] Eindhoven Institute of Renewable Energy Systems (EIRES), Eindhoven University of Technology, PO Box 513, Eindhoven 5600 MB, the Netherlands

Table of Contents

1. **Supplementary Figures**

Figure S1. Electrochemical surface area (ECSA) determination via the double-layer capacitance method.

Figure S2. Electrochemical surface area (ECSA) determination via the Pb-UPD method.

Figure S3. SEM image of the NiCu-C catalyst and accompanying EDX analysis.

Figure S4. SEM image of the synthesized carbon spheres.

Figure S5. XPS spectra of (a) Ni $2p_{3/2}$, (b) Cu $2p_{3/2}$, (c) Cu LMM, and (d) O 1s regions for Ni-C, Cu-C, NiCu-C, NiO-C, and NiO-comm samples before CO_2RR.

Figure S6. Faradaic efficiencies of products on the NiCu-C catalyst with different gas feeds: (a) He, (b) CO, and (c) CO_2 in 0.1 M KOAc solution.

Figure S7. ^{1}H-NMR results from NiCu-C in 0.1 M KOAc with (a) CO_2 and (b) CO flow, without applied bias.

Figure S8. Anderson-Schulz-Flory (ASF) plots to obtain the chain-growth probability on the Ni-C and NiCu-C catalysts.

Figure S9. Raman spectrum of Cu-C in CO_2-saturated 0.1 M $KHCO_3$ before any electrochemical measurement.

Figure S10. SEM images and corresponding EDX mapping of NiCu-C with Nafion, both before and after CO_2RR.

Figure S11. Chronoamperometry performance of NiCu-C in 0.1 M $KHCO_3$ at -0.9 V *vs.* RHE over a period of 12 hours for CO_2RR.

Figure S12-S23. Supplementary Figures: Product Analysis

Figure S24-S26. TEM analysis of the catalysts.

2. Supplementary Tables

Table S1. Atomic percentages (at%) of each element present in the Ni-C, Cu-C, and NiCu-C samples, as determined by EDX analysis.

Table S2. Ni and Cu mass loading in the Ni-C, Cu-C, and NiCu-C samples.

Table S3. Average cathodic total current density and Faradaic efficiency of all detected products from CO_2 reduction on Ni-C catalysts at different potentials.

Table S4. Average cathodic total current density and Faradaic efficiency of all detected products from CO_2 reduction on Cu-C catalysts at different potentials.

Table S5. Average cathodic total current density and Faradaic efficiency of all detected products from CO_2 reduction on NiCu-C catalysts at different potentials.

Table S6. Average cathodic total current density and Faradaic efficiency of all detected products from CO_2 reduction on NiO-C catalysts at different potentials.

Table S7. Average cathodic total current density and Faradaic efficiency of all detected products from CO_2 reduction on NiO-comm catalysts at different potentials.

Table S8. $Ni^{2+}/Ni^{\delta+}$ ratios of Ni-C, NiCu-C, NiO-C catalysts after CO_2RR.

Table S9. Average cathodic total current density and Faradaic efficiency of all detected products from CO reduction on NiCu-C catalysts at different potentials in 0.1 M $KHCO_3$ electrolyte.

Table S10. Average cathodic total current density and Faradaic efficiency of all detected products from blank experiment on NiCu-C catalysts at different potentials in 0.1 M KOAc electrolyte (He atmosphere).

Table S11. Average cathodic total current density and Faradaic efficiency of all detected products from CO reduction on NiCu-C catalysts at different potentials in 0.1 M KOAc electrolyte.

Table S12. Average cathodic total current density and Faradaic efficiency of all detected products from blank experiment on NiCu-C catalysts at different potentials in 0.1 M KOAc electrolyte.

Table S13. Measured pH values for different electrolytes.

Table S14. Average cathodic total current density and Faradaic efficiency of all detected products from CO_2 reduction on NiCu-C catalysts at different potentials in 0.1 M $KHCO_3$ + 0.05 M KOAc electrolyte.

Table S15. Average cathodic total current density and Faradaic efficiency of all detected products from CO reduction on NiCu-C catalysts at different potentials in 0.1 M $KHCO_3$ + 0.05 M KOAc electrolyte.

3. Supplementary Calculations

EDX-derived Ni and Cu Loadings.

Calculations of Crystallite Size via the Scherrer Equation

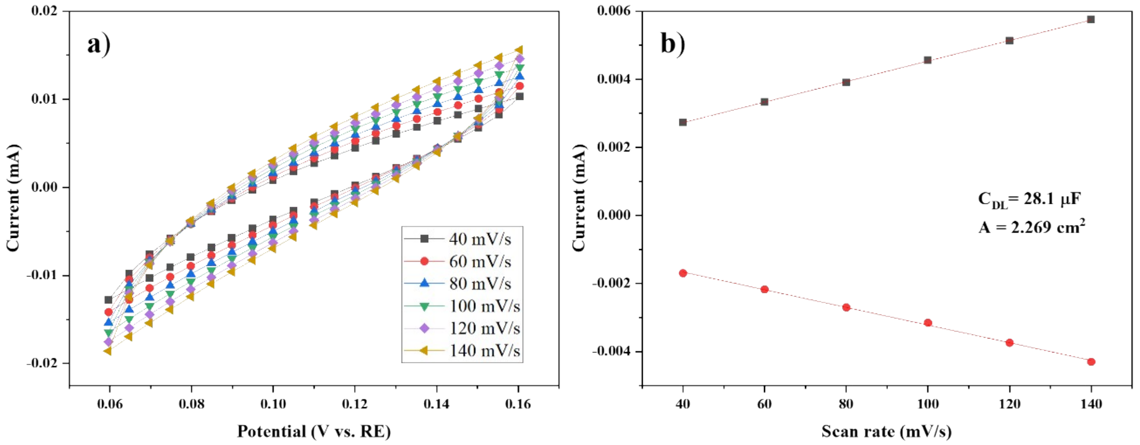

Figure S1. Electrochemical surface area (ECSA) determination via the double layer capacitance method.

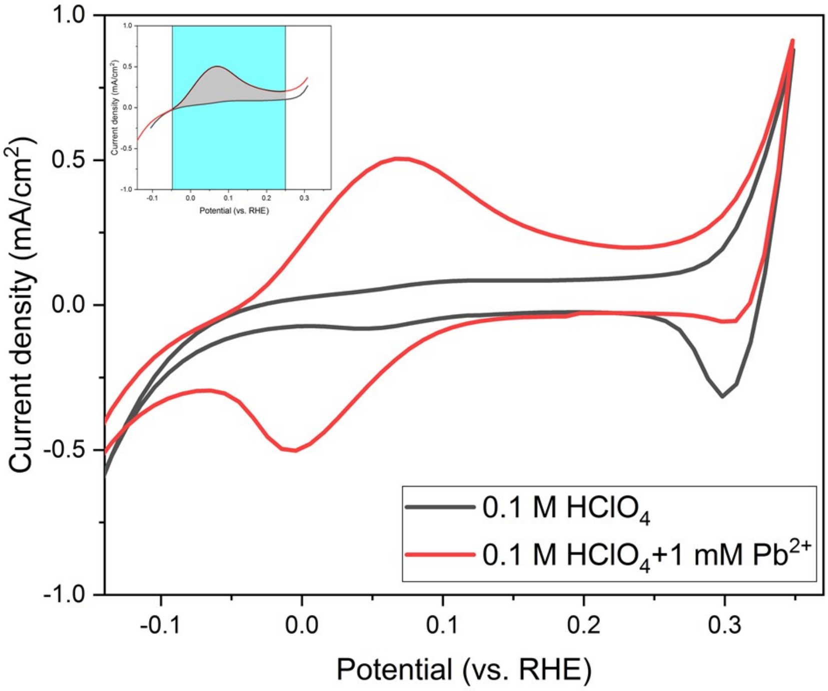

Figure S2. Electrochemical surface area (ECSA) determined via the Pb-UPD method.

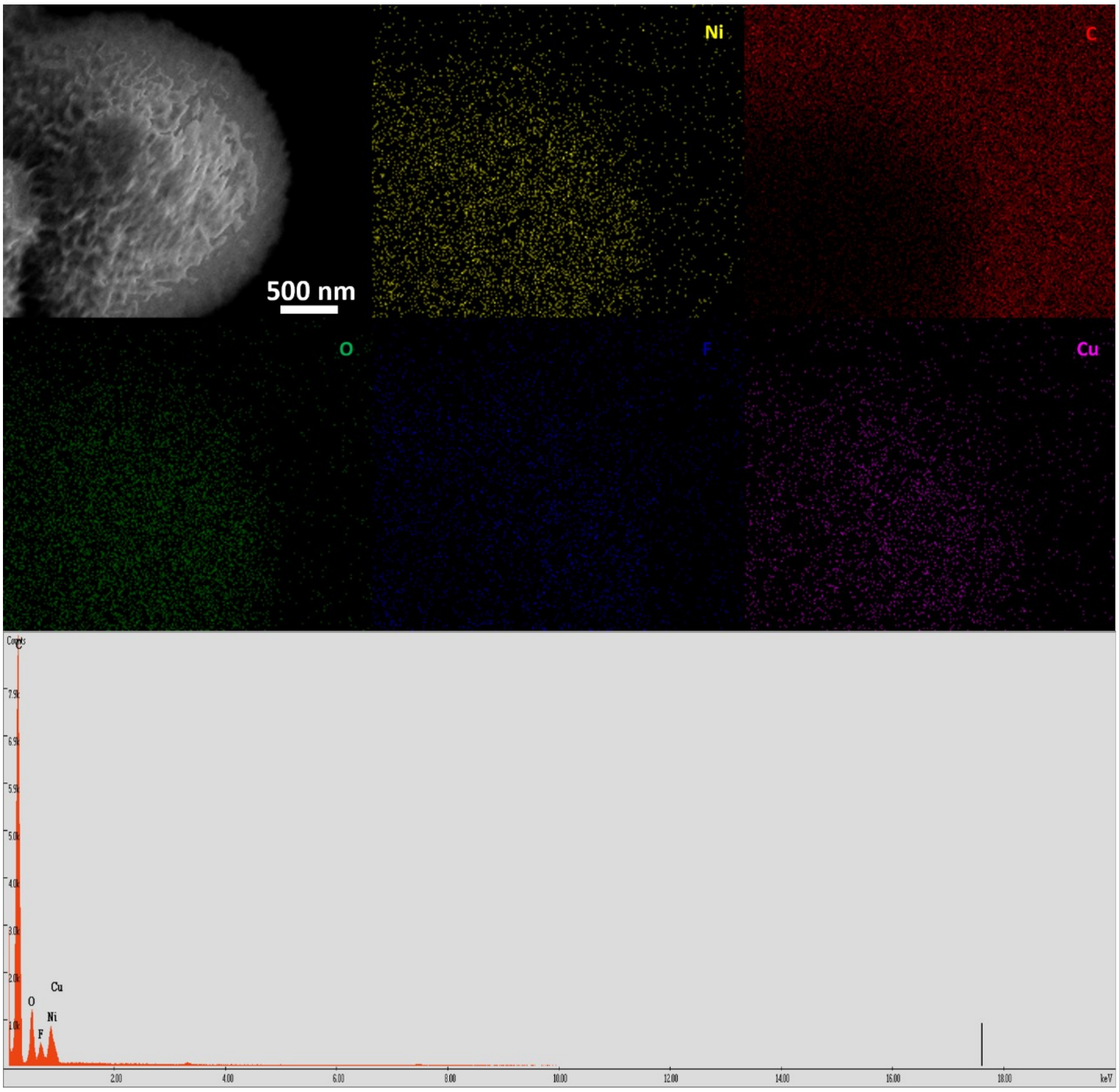

Figure S3. SEM image of NiCu-C catalyst and accompanying EDX analysis. The images show the corresponding elemental maps for nickel (Ni), carbon (C), oxygen (O), fluorine (F), and copper (Cu); the bottom panel contains the EDX spectrum. The elemental composition derived from the EDX spectrum is shown in Table S1.

Table S1. The atomic percentages (at%) of each element present in the Ni-C, Cu-C, and NiCu-C samples, as determined by EDX analysis.

Element (at%) \ Samples	Ni-C	Cu-C	NiCu-C
C_K	83.77	84.89	87.87
O_K	11.54	12.61	7.61
F_K	1.29	0.17	1.50
Ni_L	3.40		2.37
Cu_L		2.33	0.65

Ni and Cu loadings from EDX

Assuming a 1-mole sample based on the EDX atomic percentages, and considering the respective atomic weights, we can calculate the mass of each element per sample:

1) Ni-C sample

C: 83.77 At% * 1 mol * 12.01 g/mol = 10.0608 g

O: 11.54 At% * 1 mol * 16.00 g/mol = 1.8464 g

F: 1.29 At% * 1 mol * 19.00 g/mol = 0.2451 g

Ni: 3.40 At% * 1 mol * 58.69 g/mol = 1.9955 g

Total: 14.1478 g

Then, we can calculate the weight ratio of each element:

C: 10.0608 g / 14.1478 g = 71.11 wt%

O: 1.8464 g / 14.1478 g = 13.05 wt%

F: 0.2451 g / 14.1478 g = 1.73 wt%

Ni: 1.9955 g / 14.1478 g = 14.11 wt%

2) Cu-C sample

C: 84.89 At% * 1 mol * 12.01 g/mol = 10.1953 g

O: 12.61 At% * 1 mol * 16.00 g/mol = 2.0176 g

F: 0.17 At% * 1 mol * 19.00 g/mol = 0.0323 g

Cu: 2.33 At% * 1 mol * 63.55 g/mol = 1.4807 g

Total: 13.7259 g

Then, we can calculate the weight ratio of each element:

C: 10.1953 g / 13.7259 g = 74.28 wt%

O: 2.0176 g / 13.7259 g = 14.70 wt%

F: 0.0323 g / 13.7259 g = 0.24 wt%

Cu: 1.4807 g / 13.7259 g = 10.79 wt%

3) NiCu-C sample

C: 87.87 At% * 1 mol * 12.01 g/mol = 10.5532 g

O: 7.61 At% * 1 mol * 16.00 g/mol = 1.2176 g

F: 1.50 At% * 1 mol * 19.00 g/mol = 0.2850 g

Ni: 2.37 At% * 1 mol * 58.69 g/mol = 1.3909 g

Cu: 0.65 At% * 1 mol * 63.55 g/mol = 0.4131 g

Total: 13.8598 g

Then, we can calculate the weight ratio of each element:

C: 10.5532 g / 13.8598 g = 76.14 wt%

O: 1.2176 g / 13.8598 g = 8.79 wt%

F: 0.2850 g / 13.8598 g = 2.06 wt%

Ni: 1.3909 g / 13.8598 g = 10.04 wt%

Cu: 0.4131 g / 13.8598 g = 2.98 wt%

Now, we can use these weight ratios and the known catalyst loading (0.527 mg/cm^2) to find the mass loading of each element:

Ni-C:

Ni: 0.527 mg/cm^2 * 0.1411 = 74.4 μg/cm^2

Cu-C:

Cu: 0.527 mg/cm^2 * 0.1079 = 56.9 μg/cm^2

NiCu-C:

Ni: 0.527 mg/cm^2 * 0.1004 = 52.9 μg/cm^2

Cu: 0.527 mg/cm^2 * 0.0298 = 15.7 μg/cm^2

Table S2. Ni and Cu mass loading in the Ni-C, Cu-C, and NiCu-C samples.

Sample	Ni Mass Loading (μg/cm^2)	Cu Mass Loading (μg/cm^2)
Ni-C	74.4	0
Cu-C	0	56.9
NiCu-C	52.9	15.7

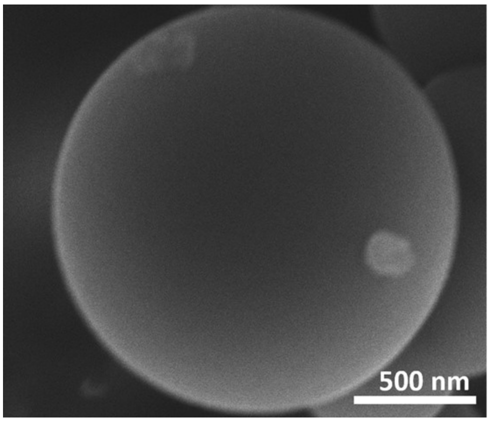

Figure S4. SEM image of the synthesized carbon sphere.

Calculations of Crystallite Size via the Scherrer Equation

The Scherrer equation is used to estimate the average crystallite size (*D*) from the broadening of XRD peaks:

$$D = K\lambda / (\beta \cos \theta)$$

Where D represents average crystallite size (in nm); K represents Scherrer constant (shape factor, typically around 0.9, but depends on the crystallite shape); λ represents X-ray wavelength (for Cu Kα radiation, λ = 0.15406 nm); β represents full width at half maximum (FWHM) of the diffraction peak (in radians); θ represents Bragg angle of the diffraction peak (in radians).

In Ni-C sample:

FWHM (β) in radians: 3° * π / 180° ≈ 0.0524 radians

Bragg angle (θ) in radians: (33.6°/2) * π / 180° ≈ 0.293 radians

Crystallite Size (D): D = (0.9 * 0.15406 nm) / (0.0524 * cos(0.293)) ≈ 2.78 nm

Note: A shape factor (K) of 0.9 was used in the Scherrer equation, which is a typical value often applied for near-spherical particles. It is important to note that this K value is an approximation in this context. The $Ni(OH)_2$ in this study forms part of a nickel fibrous structure deposited on carbon supports, and these individual $Ni(OH)_2$ nanostructures are fibrous (needle-like) rather than spherical.

Therefore, the calculated crystallite size of 2.78 nm, derived from the (110) peak, should be interpreted as an apparent crystallite size. This value primarily reflects the average diameter or width of these $Ni(OH)_2$ fibers, in the direction perpendicular to the (110) diffracting planes. The length of these fibers is likely larger and is not directly assessed by this Scherrer analysis..

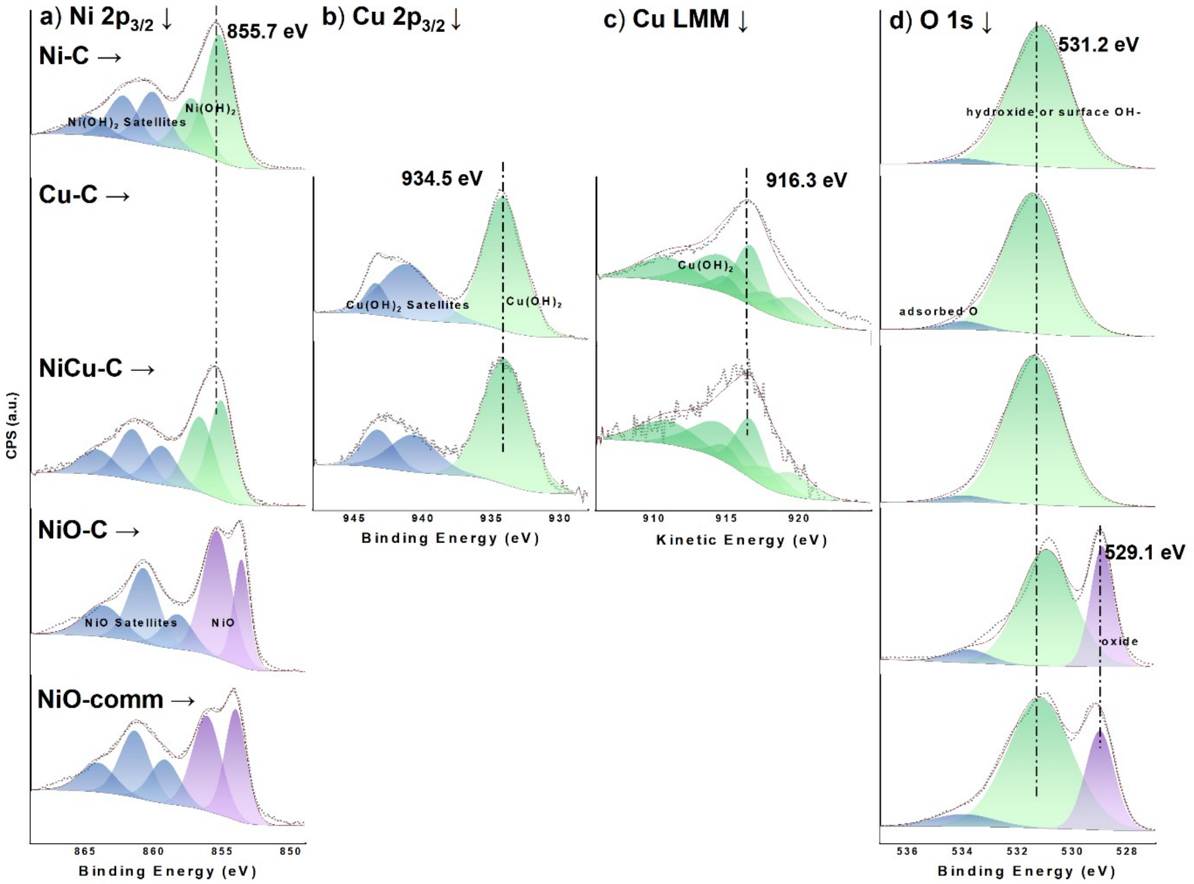

Figure S5. XPS spectra of (a) Ni $2p_{3/2}$, (b) Cu $2p_{3/2}$, (c) Cu LMM, and (d) O 1s regions for Ni-C, Cu-C, NiCu-C, NiO-C, and NiO-comm samples before CO_2RR. Blank spaces in (b) and (c) indicate the absence of a detectable Cu signal.

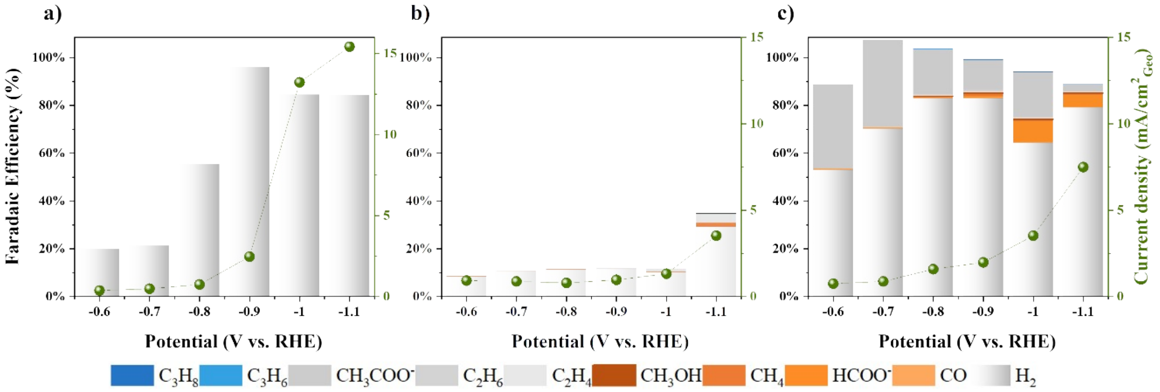

Figure S6. Faradaic efficiency of products on NiCu-C catalyst with different gases a) He, b) CO, and c) CO_2 in 0.1 M KOAc solution.

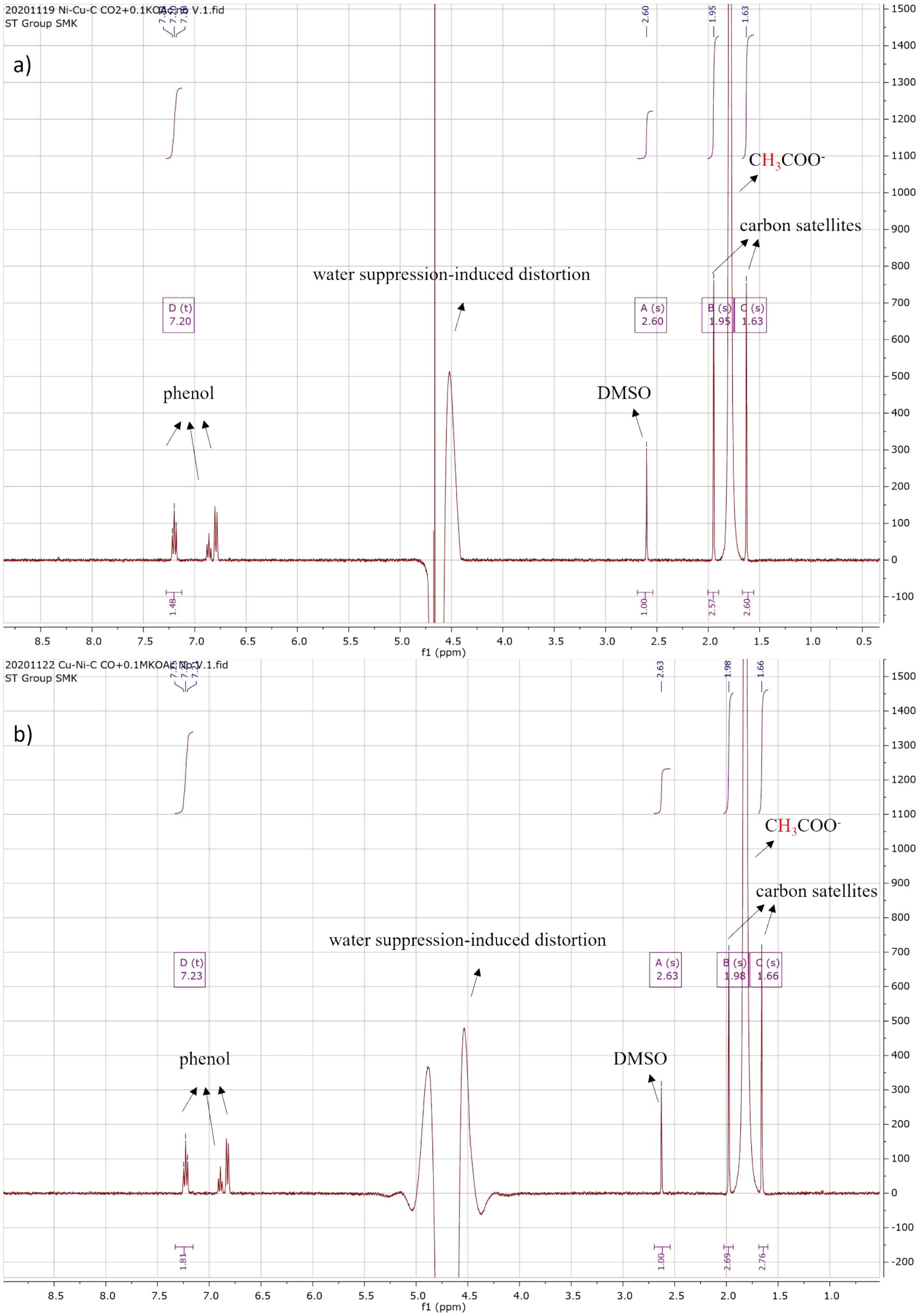

Figure S7. ^{1}H-NMR results from NiCu-C in 0.1M KOAc with a) CO_2 b) CO flow without applied bias.

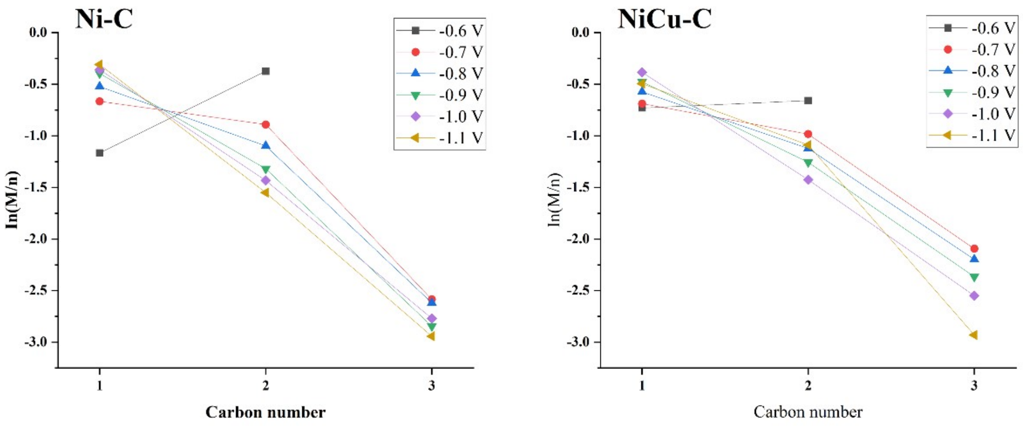

Figure S8. Anderson-Schulz-Flory plots to obtain the chain growth probability on the Ni-C and NiCu-C catalysts.

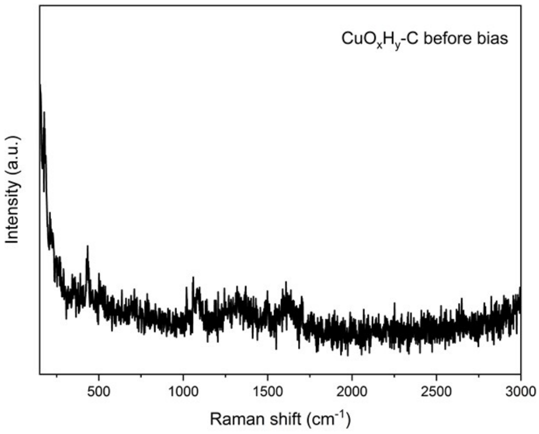

Figure S9. Raman spectrum of Cu-C in CO_2-saturated 0.1 M $KHCO_3$ before any electrochemical measurement.

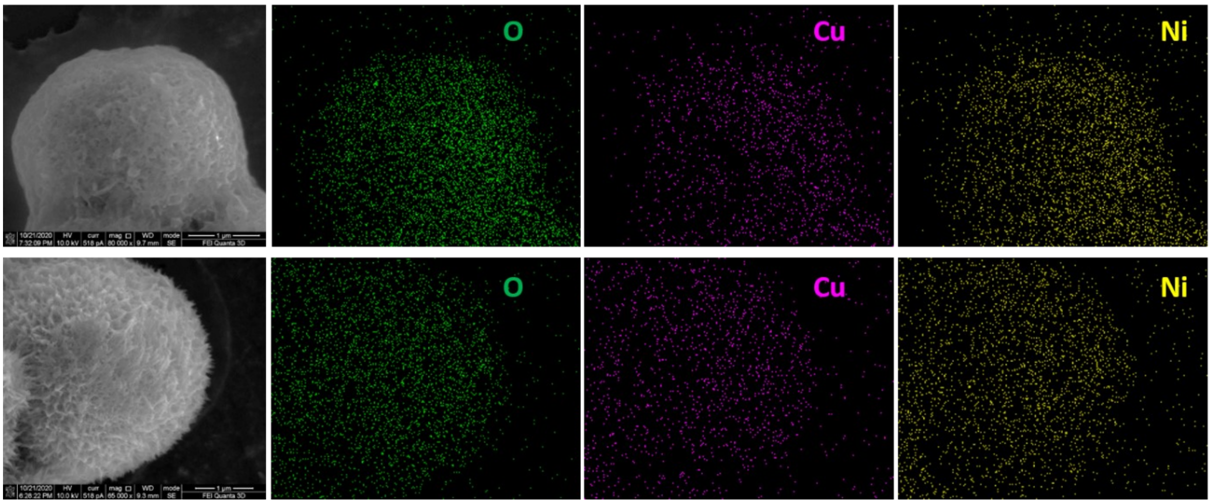

Figure S10. SEM images and corresponding EDX mapping of NiCu-C with Nafion, both before and after CO_2RR.

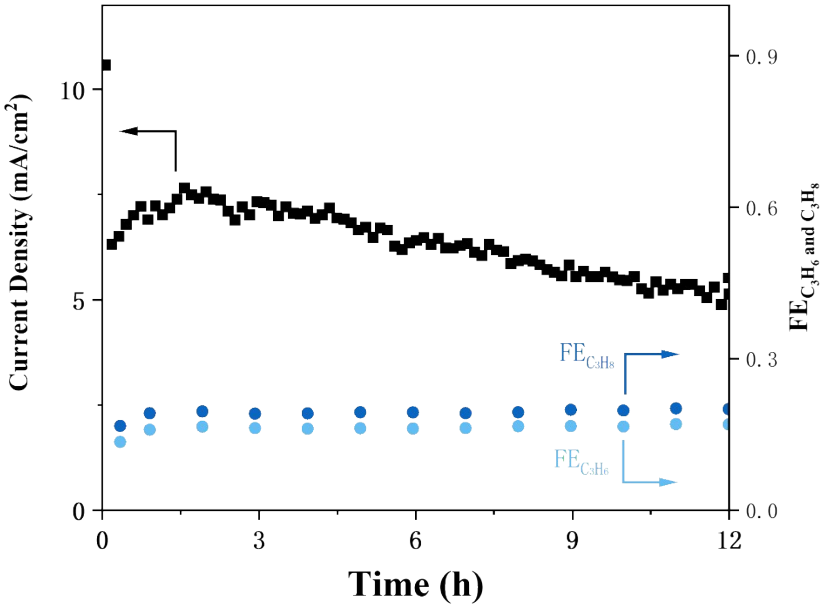

Figure S11. Chronoamperometry performance of NiCu-C in 0.1 M $KHCO_3$ at -0.9 V *vs.* RHE over a period of 12 hours for CO_2RR.

Table S3. Average cathodic total current density and Faradaic efficiency of all detected products from CO_2 reduction on Ni-C catalysts at different potentials.

Potential (V vs. RHE)	Current density (mA cm^{-2})	Faradaic efficiency (%)												
		H_2	CO	CH_4	$HCOO^-$	CH_3OH	C_2H_4	C_2H_6	CH_3COO^-	C_2H_5OH	C_3H_6	C_3H_8	C_3H_7OH	Total
-0.6	0.34	62.94	5.17	0.22	8.76	N.D.[a]	0.37	0.41	22.82	N.D.[a]	N.D.[a]	N.D.[a]	N.D.[a]	100.69
-0.7	0.48	84.75	1.91	0.90	0.89	N.D.[a]	0.36	0.84	9.66	N.D.[a]	0.13	0.18	N.D.[a]	99.63
-0.8	0.90	78.41	0.45	1.48	1.53	0.70	0.48	0.90	17.68	N.D.[a]	0.18	0.26	N.D.[a]	102.05
-0.9	1.98	87.73	0.14	2.15	0.66	0.63	0.51	0.90	3.48	N.D.[a]	0.19	0.26	N.D.[a]	96.64
-1.0	4.74	86.63	0.05	2.12	0.90	0.22	0.44	0.76	1.22	N.D.[a]	0.20	0.26	N.D.[a]	92.79
-1.1	7.87	89.75	0.02	1.99	1.41	0.24	0.34	0.61	1.61	N.D.[a]	0.15	0.19	N.D.[a]	96.32

[a] N.D.: not detected

Table S4. Average cathodic total current density and Faradaic efficiency of all detected products from CO_2 reduction on Cu-C catalysts at different potentials.

Potential (V vs. RHE)	Current density (mA cm^{-2})	Faradaic efficiency (%)												
		H_2	CO	CH_4	$HCOO^-$	CH_3OH	C_2H_4	C_2H_6	CH_3COO^-	C_2H_5OH	C_3H_6	C_3H_8	C_3H_7OH	Total
-0.6	0.58	27.43	24.73	N.D.[a]	5.71	N.D.[a]	1.39	0.21	23.65	N.D.[a]	N.D.[a]	N.D.[a]	N.D.[a]	83.12
-0.7	0.64	36.68	23.54	N.D.[a]	17.23	N.D.[a]	6.50	1.06	9.10	N.D.[a]	0.09	N.D.[a]	N.D.[a]	94.20
-0.8	1.76	27.18	12.96	N.D.[a]	10.99	N.D.[a]	12.45	0.97	3.63	19.95	0.14	N.D.[a]	3.40	91.67
-0.9	3.22	30.81	5.58	0.17	10.01	N.D.[a]	28.98	1.49	2.04	11.77	0.13	N.D.[a]	4.94	95.92
-1.0	4.85	35.21	2.09	1.20	5.28	N.D.[a]	35.23	0.72	1.30	10.92	0.05	N.D.[a]	4.40	96.41
-1.1	7.93	34.48	1.52	4.84	3.06	N.D.[a]	36.62	0.42	0.77	11.72	0.04	N.D.[a]	3.14	96.62

[a] N.D.: not detected

Table S5. Average cathodic total current density and Faradaic efficiency of all detected products from CO_2 reduction on NiCu-C catalysts at different potentials.

Potential (V vs. RHE)	Current density (mA cm^{-2})	Faradaic efficiency (%)												
		H_2	CO	CH_4	$HCOO^-$	CH_3OH	C_2H_4	C_2H_6	CH_3COO^-	C_2H_5OH	C_3H_6	C_3H_8	C_3H_7OH	Total
-0.6	0.43	57.72	1.72	0.17	2.53	N.D.[a]	0.09	0.22	6.53	N.D.[a]	N.D.[a]	N.D.[a]	N.D.[a]	68.98
-0.7	0.73	68.66	0.56	0.42	4.95	N.D.[a]	0.15	0.37	2.43	N.D.[a]	0.09	0.16	N.D.[a]	77.79
-0.8	1.42	70.72	0.60	0.85	6.32	0.36	0.28	0.53	4.13	N.D.[a]	0.17	0.23	N.D.[a]	84.18
-0.9	2.55	75.05	0.42	1.56	6.13	0.34	0.46	0.72	0.71	N.D.[a]	0.26	0.30	N.D.[a]	85.96
-1.0	5.23	85.78	0.19	2.05	6.12	0.25	0.51	0.68	0.27	N.D.[a]	0.28	0.28	N.D.[a]	96.40
-1.1	7.60	78.93	0.31	1.66	7.09	0.26	1.03	0.41	0.81	N.D.[a]	0.18	0.16	N.D.[a]	90.83

[a] N.D.: not detected

Table S6. Average cathodic total current density and Faradaic efficiency of all detected products from CO_2 reduction on NiO-C catalysts at different potentials.

Potential (V vs. RHE)	Current density (mA cm^{-2})	Faradaic efficiency (%)												
		H_2	CO	CH_4	$HCOO^-$	CH_3OH	C_2H_4	C_2H_6	CH_3COO^-	C_2H_5OH	C_3H_6	C_3H_8	C_3H_7OH	Total
-0.6	0.35	22.02	0.75	N.D.[a]	N.D.[a]	N.D.[a]	N.D.[a]	N.D.[a]	23.80	N.D.[a]	N.D.[a]	N.D.[a]	N.D.[a]	46.58
-0.7	0.53	26.34	0.52	0.19	N.D.[a]	N.D.[a]	0.05	0.18	44.18	N.D.[a]	N.D.[a]	N.D.[a]	N.D.[a]	71.45
-0.8	0.82	64.77	0.53	0.87	N.D.[a]	N.D.[a]	0.25	0.58	28.25	N.D.[a]	0.17	0.20	N.D.[a]	95.63
-0.9	1.54	83.91	0.35	1.53	1.14	N.D.[a]	0.40	0.71	10.29	N.D.[a]	0.21	0.25	N.D.[a]	98.80
-1.0	3.75	78.22	0.14	1.50	0.75	N.D.[a]	0.36	0.58	5.55	N.D.[a]	0.20	0.22	N.D.[a]	87.50
-1.1	9.26	89.45	0.05	0.41	0.20	N.D.[a]	0.10	0.15	8.22	N.D.[a]	0.06	0.06	N.D.[a]	98.69

[a] N.D.: not detected

Table S7. Average cathodic total current density and Faradaic efficiency of all detected products from CO_2 reduction on NiO-comm catalysts at different potentials.

Potential (V vs. RHE)	Current density (mA cm^{-2})	Faradaic efficiency (%)												
		H_2	CO	CH_4	$HCOO^-$	CH_3OH	C_2H_4	C_2H_6	CH_3COO^-	C_2H_5OH	C_3H_6	C_3H_8	C_3H_7OH	Total
-0.6	0.09	56.64	9.72	N.D.[a]	N.D.[a]	N.D.[a]	N.D.[a]	N.D.[a]	N.D.[a]	N.D.[a]	N.D.[a]	N.D.[a]	N.D.[a]	66.36
-0.7	0.10	74.97	6.55	0.41	N.D.[a]	N.D.[a]	N.D.[a]	N.D.[a]	N.D.[a]	N.D.[a]	N.D.[a]	N.D.[a]	N.D.[a]	81.92
-0.8	0.12	83.15	N.D.[a]	0.66	N.D.[a]	N.D.[a]	0.21	0.39	N.D.[a]	N.D.[a]	N.D.[a]	N.D.[a]	N.D.[a]	84.41
-0.9	0.29	82.23	N.D.[a]	0.68	N.D.[a]	N.D.[a]	0.23	0.27	N.D.[a]	N.D.[a]	N.D.[a]	N.D.[a]	N.D.[a]	83.42
-1.0	0.51	91.25	N.D.[a]	0.80	N.D.[a]	N.D.[a]	0.14	0.26	N.D.[a]	N.D.[a]	N.D.[a]	N.D.[a]	N.D.[a]	92.44
-1.1	1.10	91.39	N.D.[a]	0.65	N.D.[a]	N.D.[a]	0.10	0.18	N.D.[a]	N.D.[a]	0.05	0.08	N.D.[a]	92.46

[a] N.D.: not detected

Table 8. Ratio of Ni^{2+} and $Ni^{\delta+}$ in Ni-C, NiCu-C and NiO-C samples after CO_2 reduction obtained by analysis of XPS data.

Samples	Area of Ni^{2+}/CPS (a.u.)	Area of $Ni^{\delta+}$	Ni^{2+} : $Ni^{\delta+}$
Ni-C	138739.6	22328.3	0.86 : 0.14
NiCu-C	42972.6	4263.8	0.91 : 0.09
NiO-C	70233.3	3787.1	0.95 : 0.05

Table S9. Average cathodic total current density and Faradaic efficiency of all detected products from CO reduction on NiCu-C catalysts at different potentials in 0.1 M $KHCO_3$ electrolyte.

Potential (V vs. RHE)	Current density (mA cm^{-2})	Faradaic efficiency (%)												
		H_2	CO	CH_4	$HCOO^-$	CH_3OH	C_2H_4	C_2H_6	CH_3COO^-	C_2H_5OH	C_3H_6	C_3H_8	C_3H_7OH	Total
-0.6	1.06	5.77	N.D.[a]	0.10	N.D.[a]	N.D.[a]	N.D.[a]	N.D.[a]	37.05	N.D.[a]	N.D.[a]	N.D.[a]	N.D.[a]	42.93
-0.7	1.15	7.64	N.D.[a]	0.10	N.D.[a]	N.D.[a]	0.02	0.03	25.53	N.D.[a]	N.D.[a]	N.D.[a]	N.D.[a]	33.32
-0.8	1.15	9.64	N.D.[a]	0.12	N.D.[a]	N.D.[a]	0.05	0.03	9.91	N.D.[a]	0.03	0.03	N.D.[a]	19.80
-0.9	1.54	7.72	N.D.[a]	0.13	N.D.[a]	N.D.[a]	0.18	0.07	4.73	N.D.[a]	0.03	0.03	N.D.[a]	12.93
-1.0	2.86	11.68	N.D.[a]	0.24	N.D.[a]	N.D.[a]	1.18	0.05	2.75	N.D.[a]	0.07	0.07	N.D.[a]	16.01
-1.1	6.61	42.51	N.D.[a]	2.42	0.26	N.D.[a]	1.75	0.03	3.65	N.D.[a]	0.05	0.05	N.D.[a]	50.46

[a] N.D.: not detected

Table 10. Average cathodic total current density and Faradaic efficiency of all detected products from blank experiment on NiCu-C catalysts at different potentials in 0.1 M KOAc electrolyte (He atmosphere).

Potential (V vs. RHE)	Current density (mA cm^{-2})	Faradaic efficiency (%)												
		H_2	CO	CH_4	$HCOO^-$	CH_3OH	C_2H_4	C_2H_6	CH_3COO^-	C_2H_5OH	C_3H_6	C_3H_8	C_3H_7OH	Total
-0.6	0.37	19.70	N.D.[a]	N.D.[a]	N.D.[a]	N.D.[a]	N.D.[a]	N.D.[a]	N.D.[a]	N.D.[a]	N.D.[a]	N.D.[a]	N.D.[a]	19.70
-0.7	0.48	21.15	N.D.[a]	N.D.[a]	N.D.[a]	N.D.[a]	N.D.[a]	N.D.[a]	N.D.[a]	N.D.[a]	N.D.[a]	N.D.[a]	N.D.[a]	21.15
-0.8	0.75	55.36	N.D.[a]	N.D.[a]	N.D.[a]	N.D.[a]	N.D.[a]	N.D.[a]	N.D.[a]	N.D.[a]	N.D.[a]	N.D.[a]	N.D.[a]	55.36
-0.9	2.47	95.92	N.D.[a]	N.D.[a]	N.D.[a]	N.D.[a]	N.D.[a]	N.D.[a]	N.D.[a]	N.D.[a]	N.D.[a]	N.D.[a]	N.D.[a]	95.92
-1.0	13.22	84.49	N.D.[a]	N.D.[a]	N.D.[a]	N.D.[a]	N.D.[a]	N.D.[a]	N.D.[a]	N.D.[a]	N.D.[a]	N.D.[a]	N.D.[a]	84.49
-1.1	15.43	84.24	N.D.[a]	N.D.[a]	N.D.[a]	N.D.[a]	N.D.[a]	N.D.[a]	N.D.[a]	N.D.[a]	N.D.[a]	N.D.[a]	N.D.[a]	84.24

[a] N.D.: not detected

Table S11. Average cathodic total current density and Faradaic efficiency of all detected products from CO reduction on NiCu-C catalysts at different potentials in 0.1 M KOAc electrolyte.

Potential (V vs. RHE)	Current density (mA cm^{-2})	Faradaic efficiency (%)												
		H_2	CO	CH_4	$HCOO^-$	CH_3OH	C_2H_4	C_2H_6	CH_3COO^-	C_2H_5OH	C_3H_6	C_3H_8	C_3H_7OH	Total
-0.6	0.93	8.10	N.D.[a]	0.31	N.D.[a]	N.D.[a]	N.D.[a]	N.D.[a]	N.D.[a]	N.D.[a]	N.D.[a]	N.D.[a]	N.D.[a]	8.41
-0.7	0.88	10.54	N.D.[a]	0.28	N.D.[a]	N.D.[a]	N.D.[a]	N.D.[a]	N.D.[a]	N.D.[a]	N.D.[a]	N.D.[a]	N.D.[a]	10.82
-0.8	0.79	11.15	N.D.[a]	0.28	N.D.[a]	N.D.[a]	0.04	N.D.[a]	N.D.[a]	N.D.[a]	N.D.[a]	N.D.[a]	N.D.[a]	11.48
-0.9	0.97	11.38	N.D.[a]	0.23	N.D.[a]	N.D.[a]	0.22	N.D.[a]	N.D.[a]	N.D.[a]	0.02	N.D.[a]	N.D.[a]	11.84
-1.0	1.32	10.10	N.D.[a]	0.21	N.D.[a]	N.D.[a]	1.21	0.03	N.D.[a]	N.D.[a]	0.02	N.D.[a]	N.D.[a]	11.57
-1.1	3.53	28.91	N.D.[a]	1.55	0.26	N.D.[a]	3.89	0.03	N.D.[a]	N.D.[a]	0.04	0.01	N.D.[a]	34.68

[a] N.D.: not detected

Table S12. Average cathodic total current density and Faradaic efficiency of all detected products from CO_2 reduction on NiCu-C catalysts at different potentials in 0.1 M KOAc electrolyte.

Potential (V vs. RHE)	Current density (mA cm^{-2})	Faradaic efficiency (%)												
		H_2	CO	CH_4	$HCOO^-$	CH_3OH	C_2H_4	C_2H_6	CH_3COO^-	C_2H_5OH	C_3H_6	C_3H_8	C_3H_7OH	Total
-0.6	0.75	52.89	0.47	0.04	N.D.[a]	N.D.[a]	0.03	0.05	35.15	N.D.[a]	N.D.[a]	N.D.[a]	N.D.[a]	88.63
-0.7	0.88	70.11	0.47	0.18	N.D.[a]	N.D.[a]	0.08	0.18	36.38	N.D.[a]	N.D.[a]	N.D.[a]	N.D.[a]	107.40
-0.8	1.59	82.69	0.58	0.56	N.D.[a]	0.22	0.15	0.36	19.02	N.D.[a]	0.11	0.19	N.D.[a]	103.88
-0.9	1.98	82.99	0.41	1.23	0.40	0.37	0.40	0.76	12.30	N.D.[a]	0.20	0.32	N.D.[a]	99.38
-1.0	3.53	64.30	0.25	0.57	8.92	0.52	0.28	0.31	18.70	N.D.[a]	0.10	0.12	N.D.[a]	94.06
-1.1	7.49	78.97	0.20	0.82	5.06	0.22	0.36	0.32	2.65	N.D.[a]	0.13	0.13	N.D.[a]	88.88

[a] N.D.: not detected

Table 13. Measured pH values for different electrolytes.

Electrolytes	Conditions	pH
0.1 M $KHCO_3$	CO_2 saturated	6.8
0.1 M $KHCO_3$	fresh prepared / CO saturated	8.3
0.1 M KOAc	CO_2 saturated	5.8
0.1 M KOAc	fresh prepared / CO saturated	7.2
0.1 M $KHCO_3$ + 0.05 M KOAc	CO_2 saturated	6.8
0.1 M $KHCO_3$ + 0.05 M KOAc	fresh prepared / CO saturated	8.5

Table S14. Average cathodic total current density and Faradaic efficiency of all detected products from CO_2 reduction on NiCu-C catalysts at different potentials in 0.1 M $KHCO_3$ + 0.05 M KOAc electrolyte.

Potential (V vs. RHE)	Current density (mA cm^{-2})	Faradaic efficiency (%)												
		H_2	CO	CH_4	$HCOO^-$	CH_3OH	C_2H_4	C_2H_6	CH_3COO^-	C_2H_5OH	C_3H_6	C_3H_8	C_3H_7OH	Total
-0.6	0.62	54.66	3.96	0.13	0.64	N.D.[a]	0.05	0.14	16.72	N.D.[a]	N.D.[a]	N.D.[a]	N.D.[a]	76.30
-0.7	0.79	53.59	1.28	0.28	5.09	N.D.[a]	0.11	0.33	13.74	N.D.[a]	0.08	0.19	N.D.[a]	74.68
-0.8	1.59	66.18	0.57	0.92	5.31	N.D.[a]	0.25	0.65	2.59	N.D.[a]	0.13	0.19	N.D.[a]	76.79
-0.9	2.20	69.29	0.84	1.28	7.06	0.85	0.60	0.70	3.30	N.D.[a]	0.27	0.32	N.D.[a]	84.50
-1.0	5.95	74.86	0.38	1.77	9.28	0.41	0.54	0.64	1.56	N.D.[a]	0.30	0.28	N.D.[a]	90.02
-1.1	11.46	82.07	0.13	1.37	5.58	0.32	0.37	0.41	0.64	N.D.[a]	0.20	0.17	N.D.[a]	91.27

[a] N.D.: not detected

Table S15. Average cathodic total current density and Faradaic efficiency of all detected products from CO reduction on NiCu-C catalysts at different potentials in 0.1 M $KHCO_3$ + 0.05 M KOAc electrolyte.

Potential (V vs. RHE)	Current density (mA cm^{-2})	Faradaic efficiency (%)												
		H_2	CO	CH_4	$HCOO^-$	CH_3OH	C_2H_4	C_2H_6	CH_3COO^-	C_2H_5OH	C_3H_6	C_3H_8	C_3H_7OH	Total
-0.6	1.01	4.05	N.D.[a]	0.02	N.D.[a]	N.D.[a]	N.D.[a]	N.D.[a]	N.D.[a]	N.D.[a]	0.01	N.D.[a]	N.D.[a]	4.08
-0.7	0.93	5.43	N.D.[a]	0.07	N.D.[a]	N.D.[a]	0.04	0.04	N.D.[a]	N.D.[a]	0.02	N.D.[a]	N.D.[a]	5.60
-0.8	1.15	11.87	N.D.[a]	0.10	N.D.[a]	N.D.[a]	0.15	0.04	N.D.[a]	N.D.[a]	0.04	0.03	N.D.[a]	12.23
-0.9	1.72	8.43	N.D.[a]	0.10	N.D.[a]	N.D.[a]	0.67	0.05	N.D.[a]	N.D.[a]	0.04	0.02	N.D.[a]	9.31
-1.0	3.26	21.70	N.D.[a]	0.40	N.D.[a]	N.D.[a]	3.60	0.03	N.D.[a]	N.D.[a]	0.05	0.01	N.D.[a]	25.80
-1.1	10.14	72.35	N.D.[a]	2.11	N.D.[a]	N.D.[a]	1.02	0.01	N.D.[a]	N.D.[a]	0.03	0.00	N.D.[a]	75.53

[a] N.D.: not detected

Supplementary Figures: Product Analysis

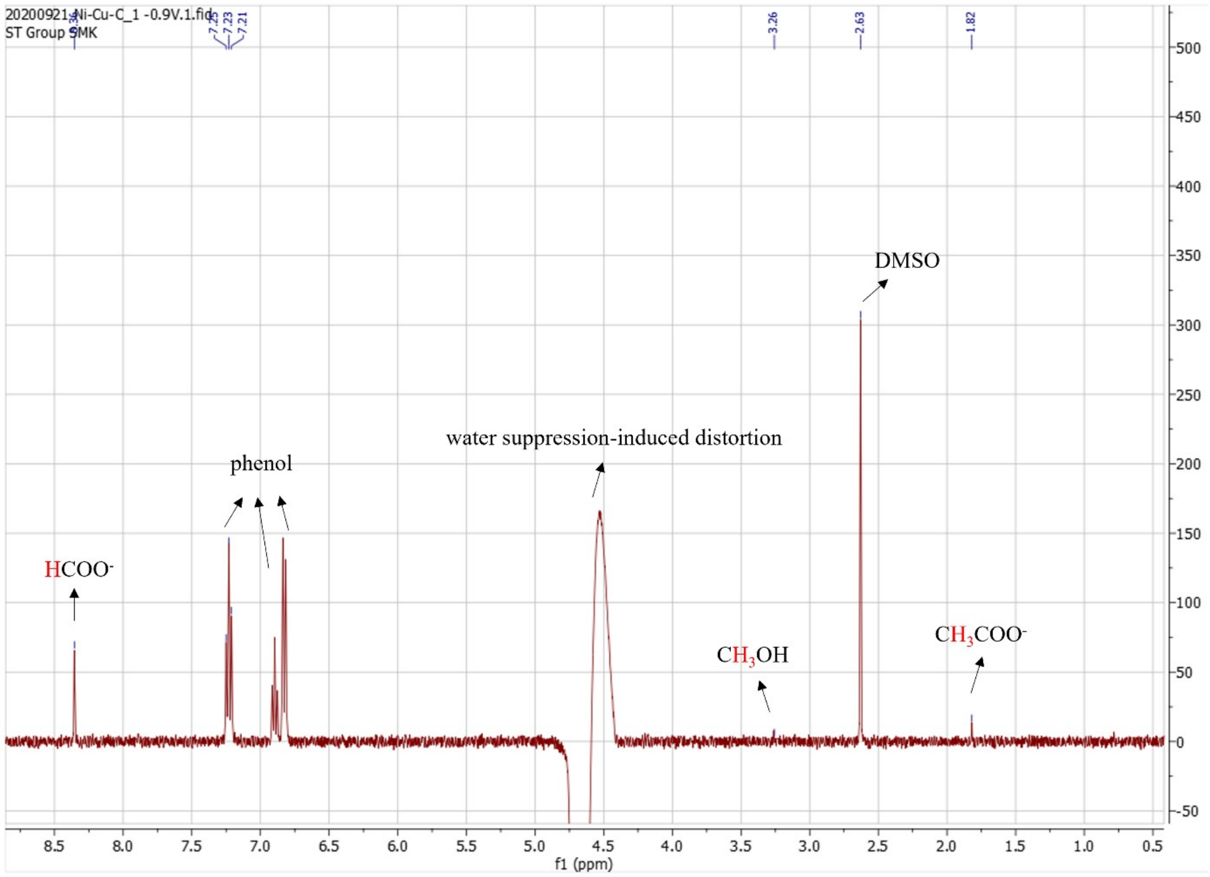

Figure S12. Representative ^{1}H NMR spectrum of the catholyte from one of the independent CO_2 reduction experiments on NiCu-C catalyst (CO_2RR at -0.9 V vs. RHE in 0.1 M $KHCO_3$).

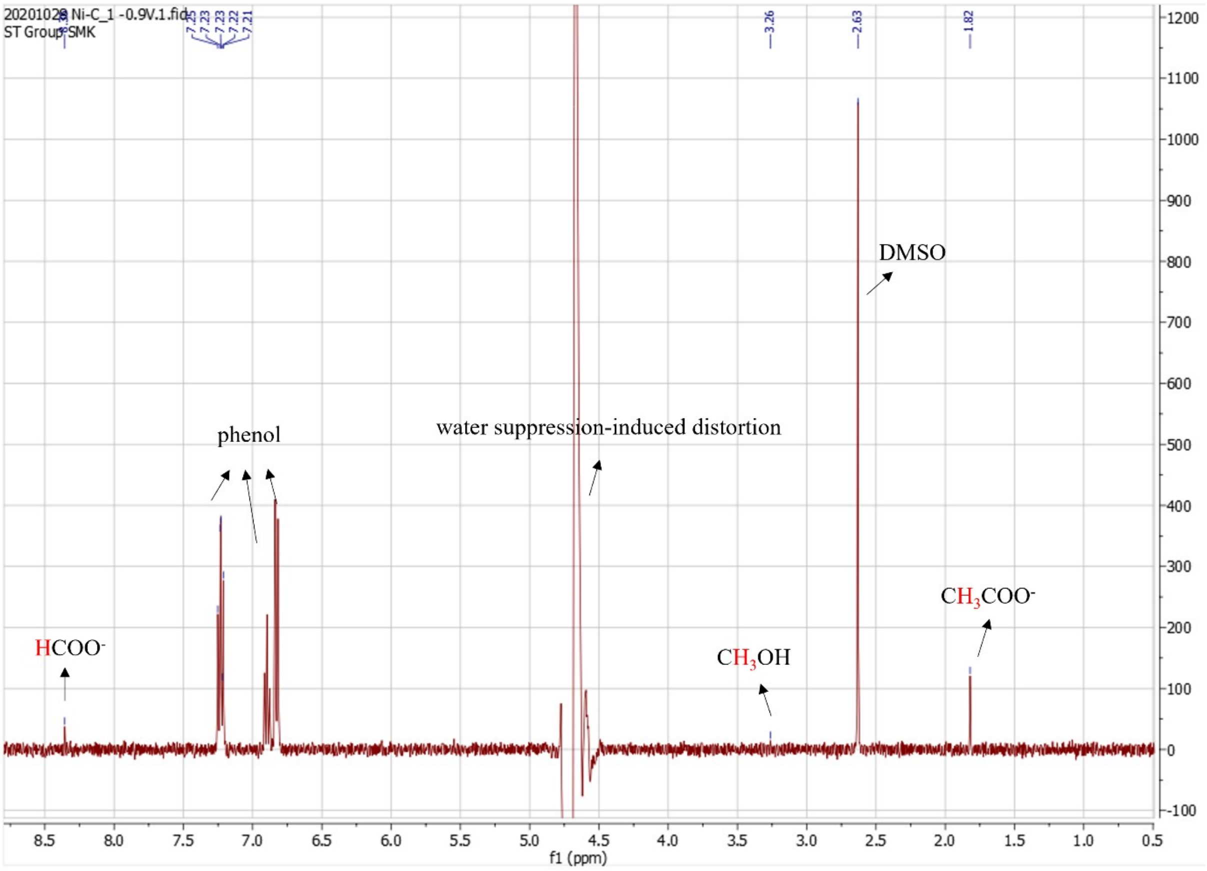

Figure S13. Representative ^{1}H NMR spectrum of the catholyte from one of the independent CO_2 reduction experiments on Ni-C catalyst (CO_2RR at -0.9 V vs. RHE in 0.1 M $KHCO_3$).

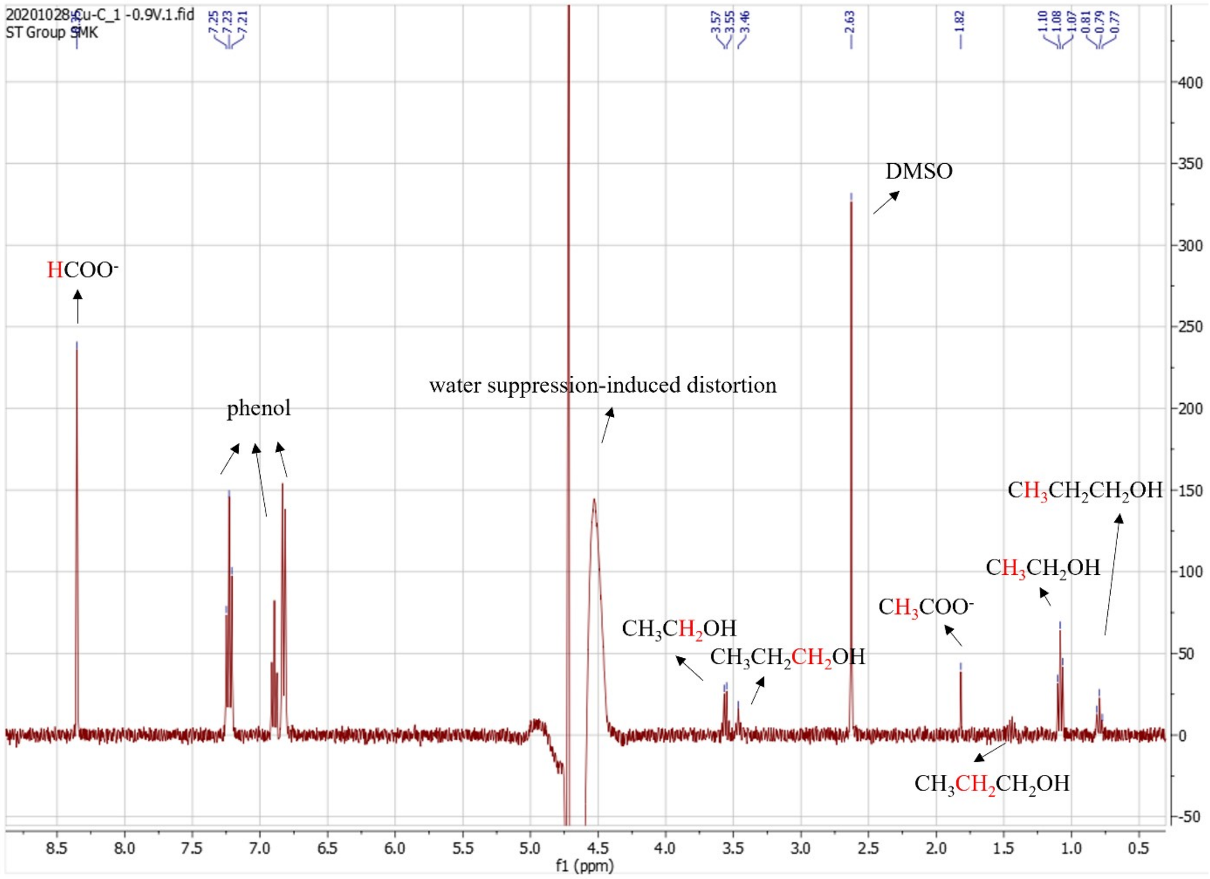

Figure S14. Representative ^{1}H NMR spectrum of the catholyte from one of the independent CO_2 reduction experiments on Cu-C catalyst (CO_2RR at -0.9 V vs. RHE in 0.1 M $KHCO_3$).

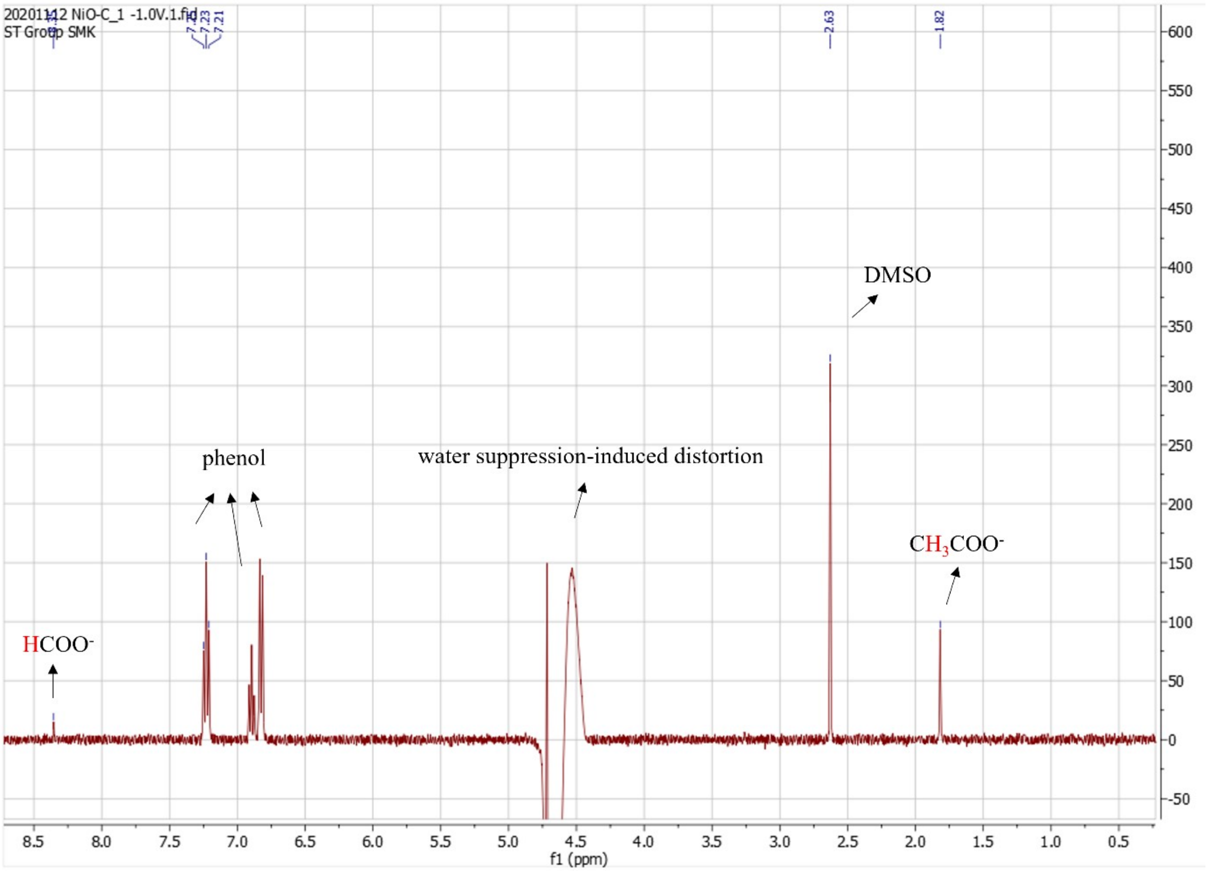

Figure S15. Representative ^{1}H NMR spectrum of the catholyte from one of the independent CO_2 reduction experiments on NiO-C catalyst (CO_2RR at -1.0 V vs. RHE in 0.1 M $KHCO_3$).

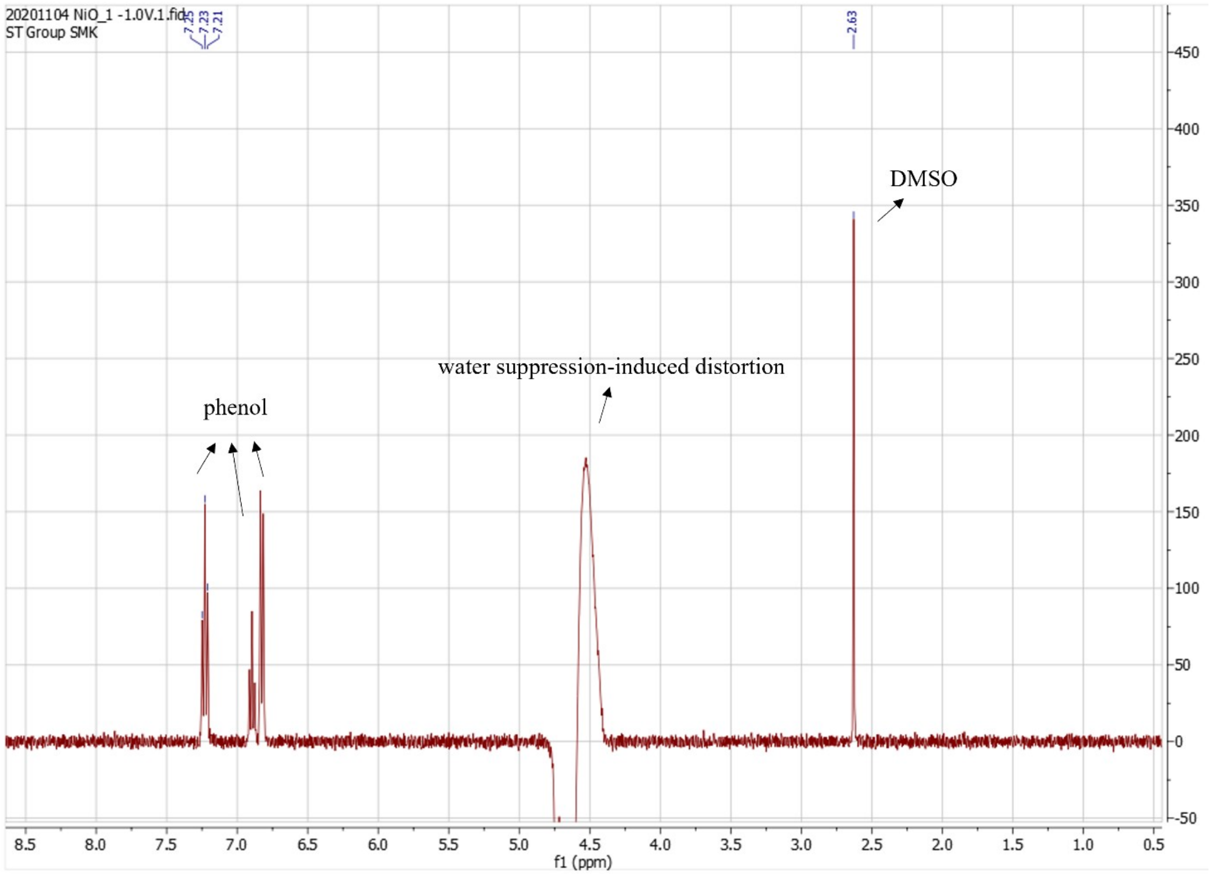

Figure S16. Representative ^{1}H NMR spectrum of the catholyte from one of the independent CO_2 reduction experiments on commercial NiO catalyst (CO_2RR at -1.0 V vs. RHE in 0.1 M $KHCO_3$).

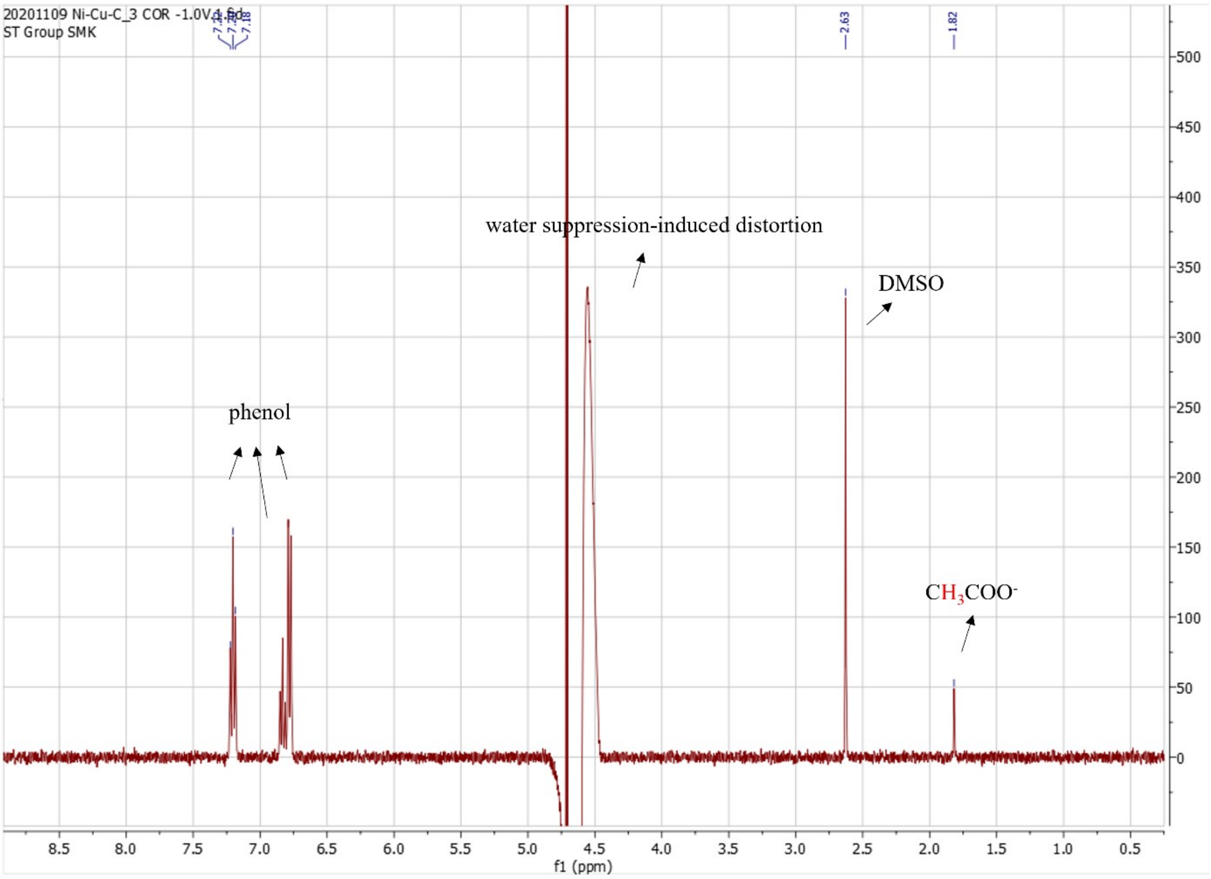

Figure S17. Representative ^1H NMR spectrum of the catholyte from one of the independent CO_2 reduction experiments on NiCu-C catalyst (CORR at -1.0 V vs. RHE in 0.1 M $KHCO_3$).

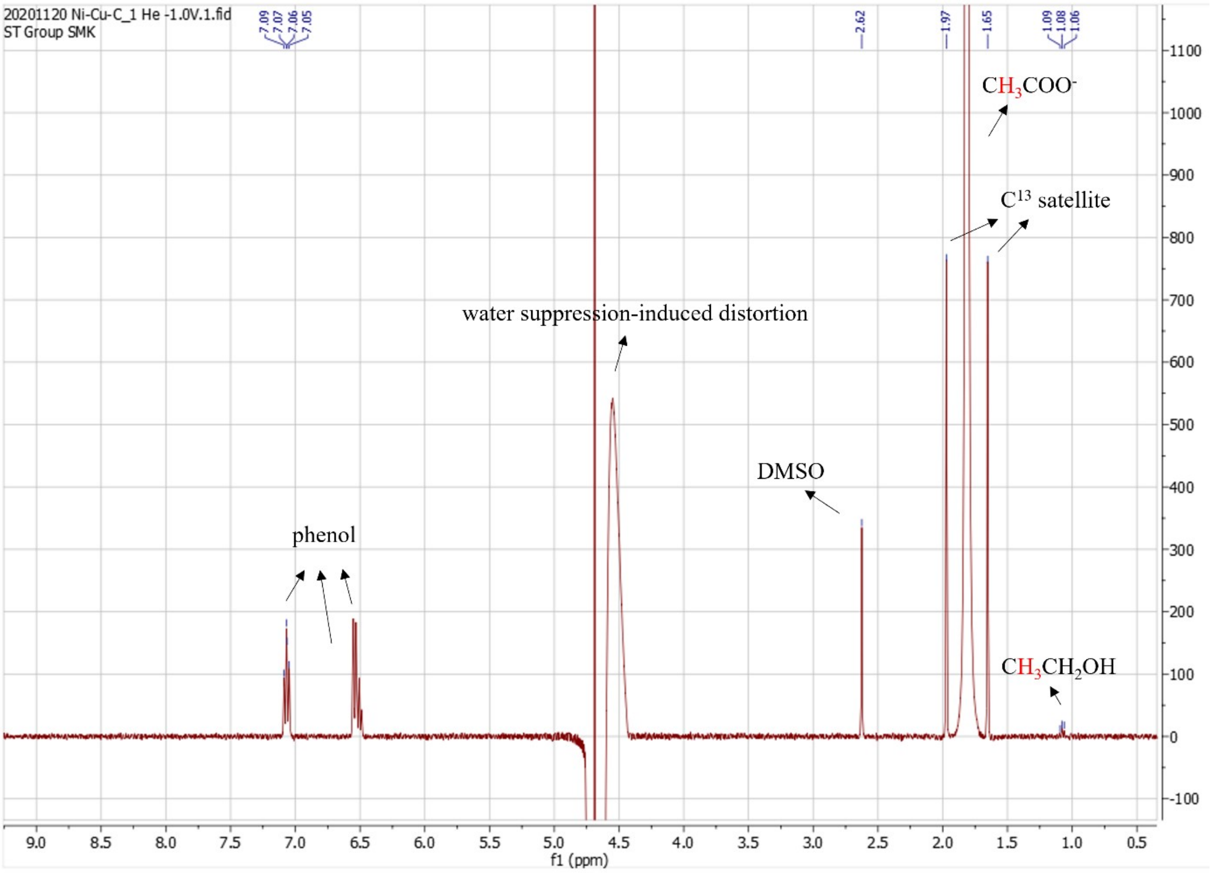

Figure S18. Representative ^{1}H NMR spectrum of the catholyte from one of the independent CO_2 reduction experiments on NiCu-C catalyst (helium atmosphere at -1.0 V vs. RHE in 0.1 M KOAc).

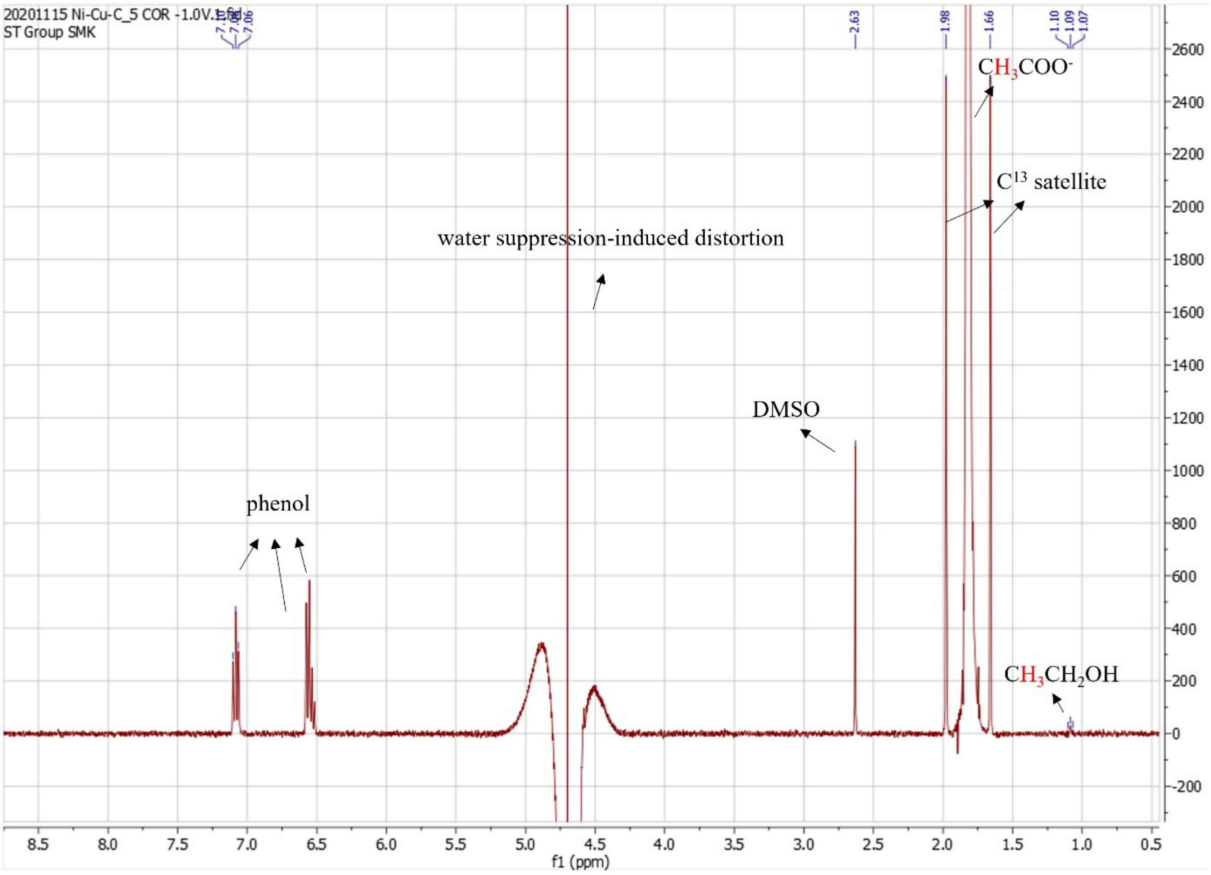

Figure S19. Representative ^{1}H NMR spectrum of the catholyte from one of the independent CO_2 reduction experiments on NiCu-C catalyst (CORR at -1.0 V vs. RHE in 0.1 M KOAc).

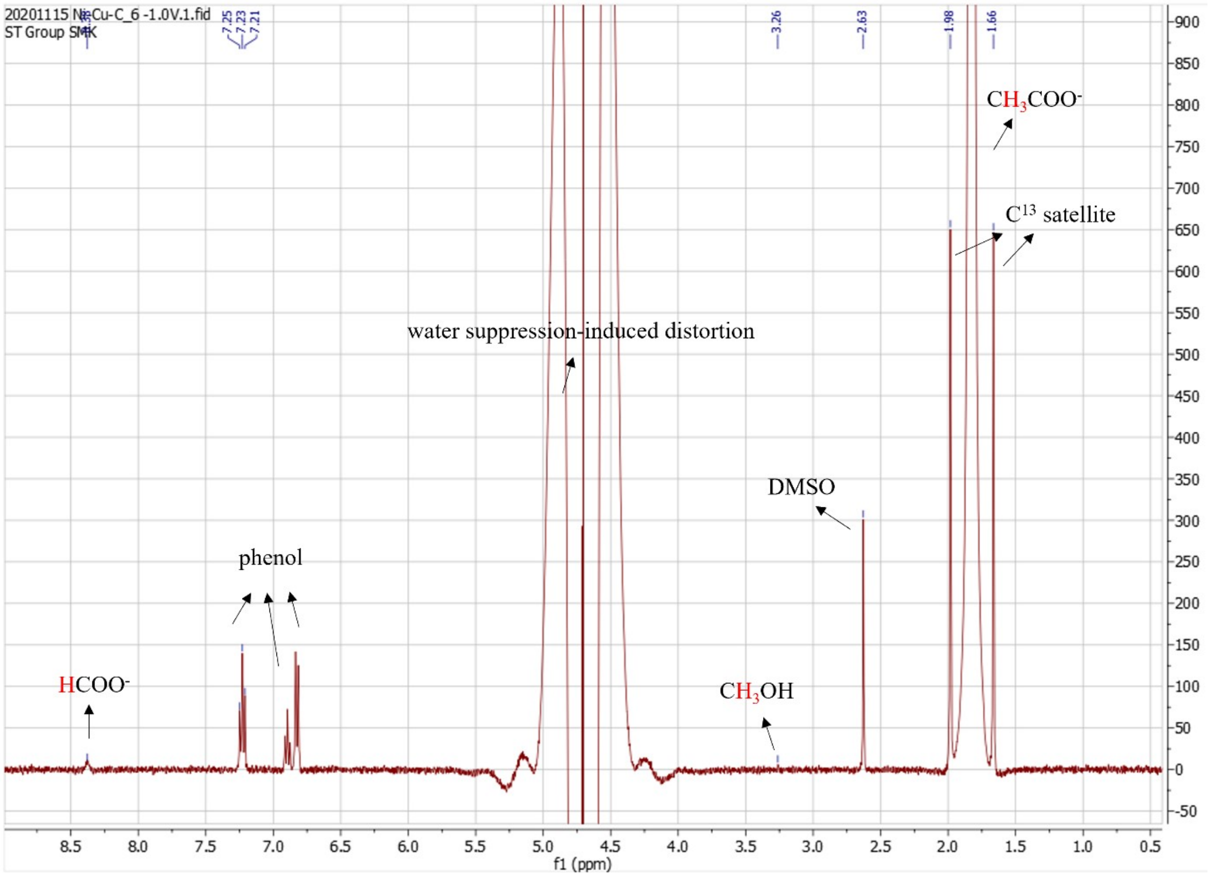

Figure S20. Representative ^{1}H NMR spectrum of the catholyte from one of the independent CO_2 reduction experiments on NiCu-C catalyst (CO_2RR at -1.0 V vs. RHE in 0.1 M KOAc).

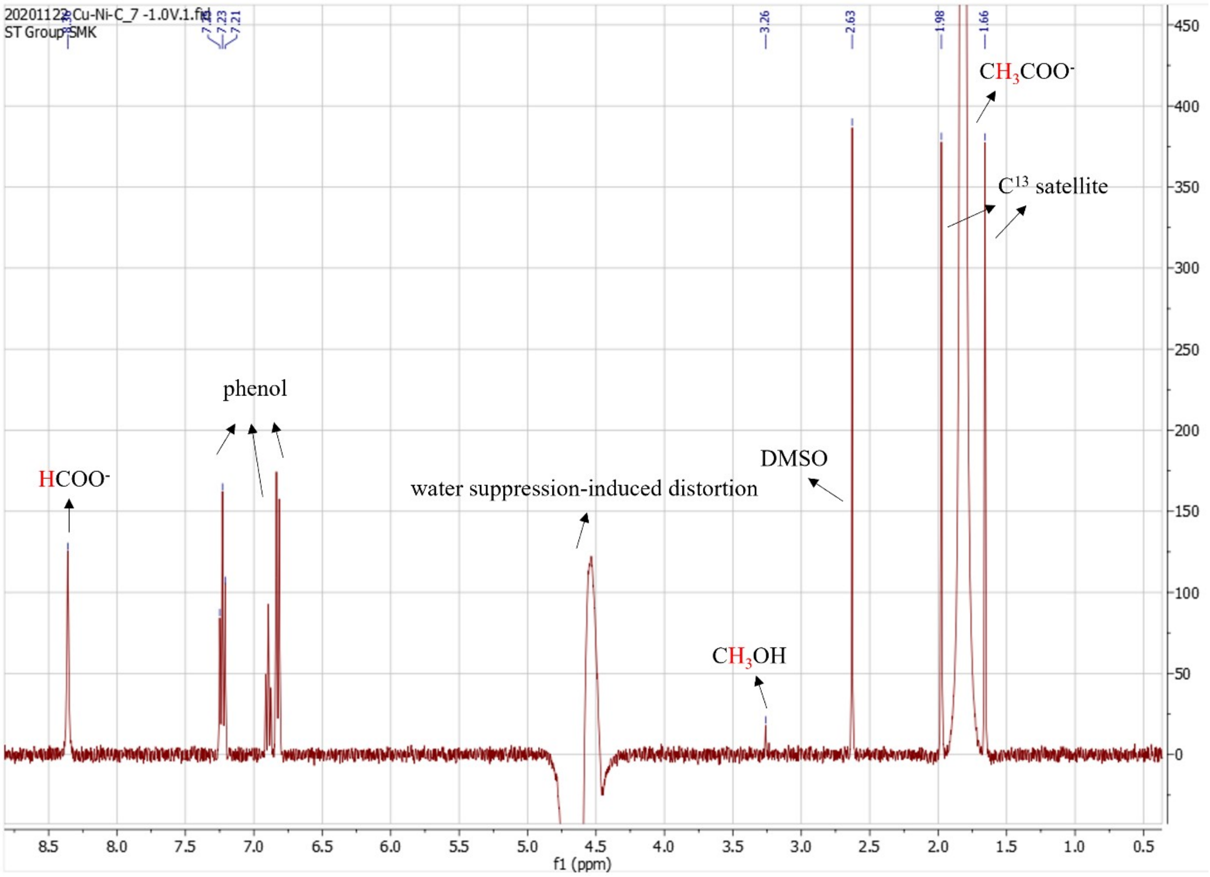

Figure S21. Representative ^{1}H NMR spectrum of the catholyte from one of the independent CO_2 reduction experiments on NiCu-C catalyst (CO_2RR at -1.0 V vs. RHE in 0.1 M KHCO3+0.05 M KOAc).

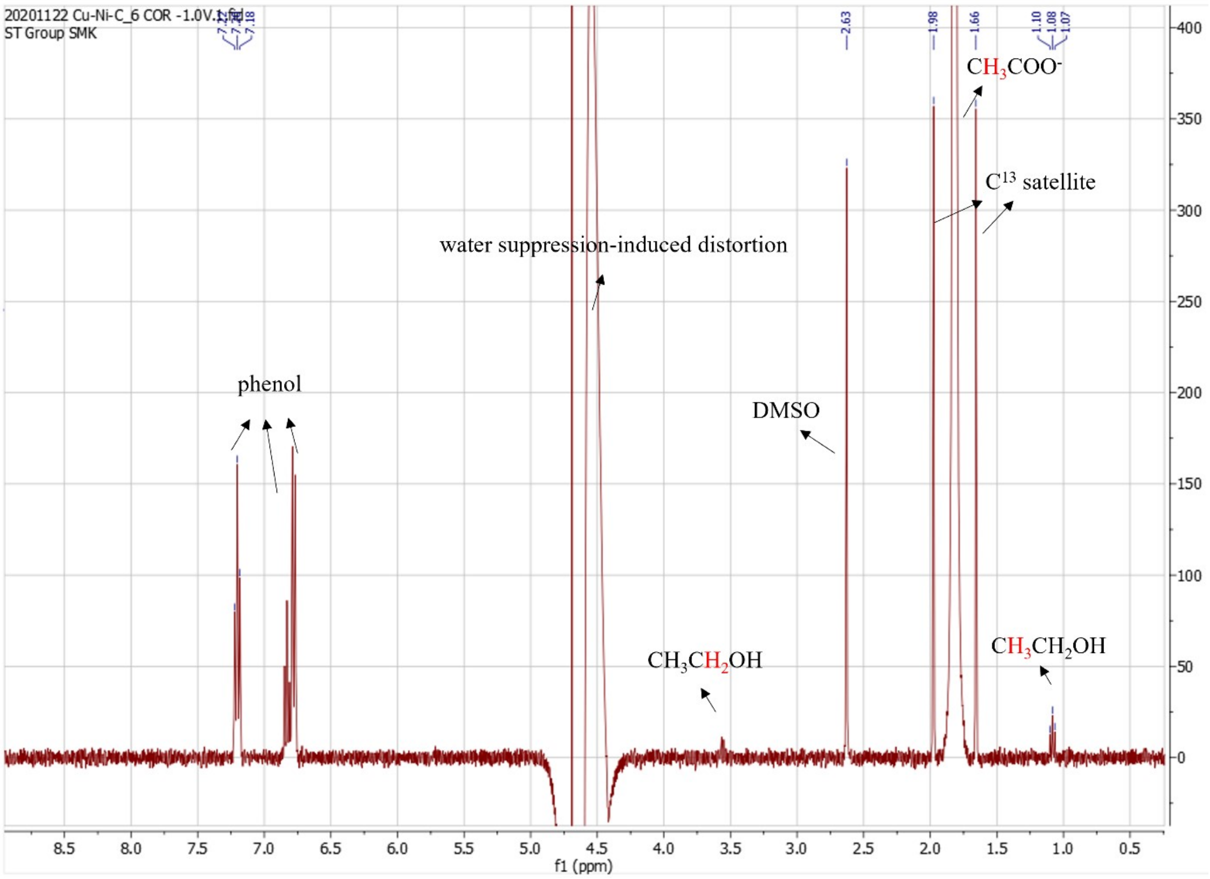

Figure S22. Representative ^{1}H NMR spectrum of the catholyte from one of the independent CO_2 reduction experiments on NiCu-C catalyst (CORR at -1.0 V vs. RHE in 0.1 M KHCO3+0.05 M KOAc).

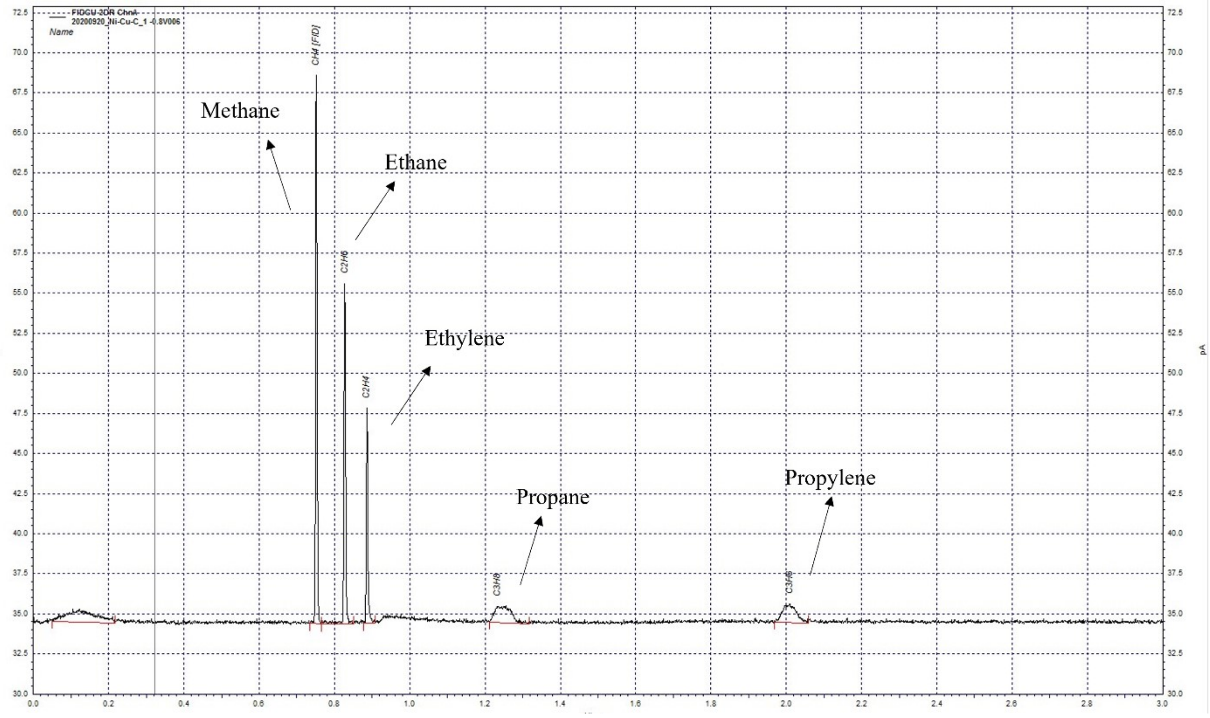

Figure S23. Representative GC signal of gas products from one of the independent CO_2 reduction experiments on NiCu-C catalyst (CO_2RR at -0.8 V vs. RHE in 0.1 M $KHCO_3$).

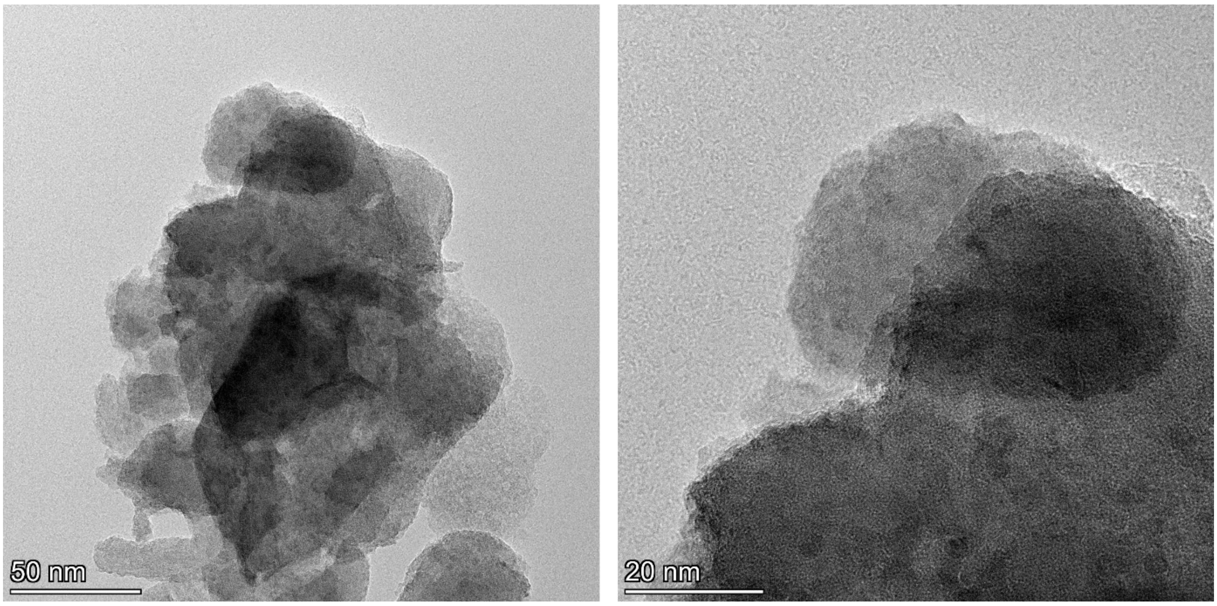

Figure S24. Bright-Field TEM (BFTEM) image of Ni-C catalyst.

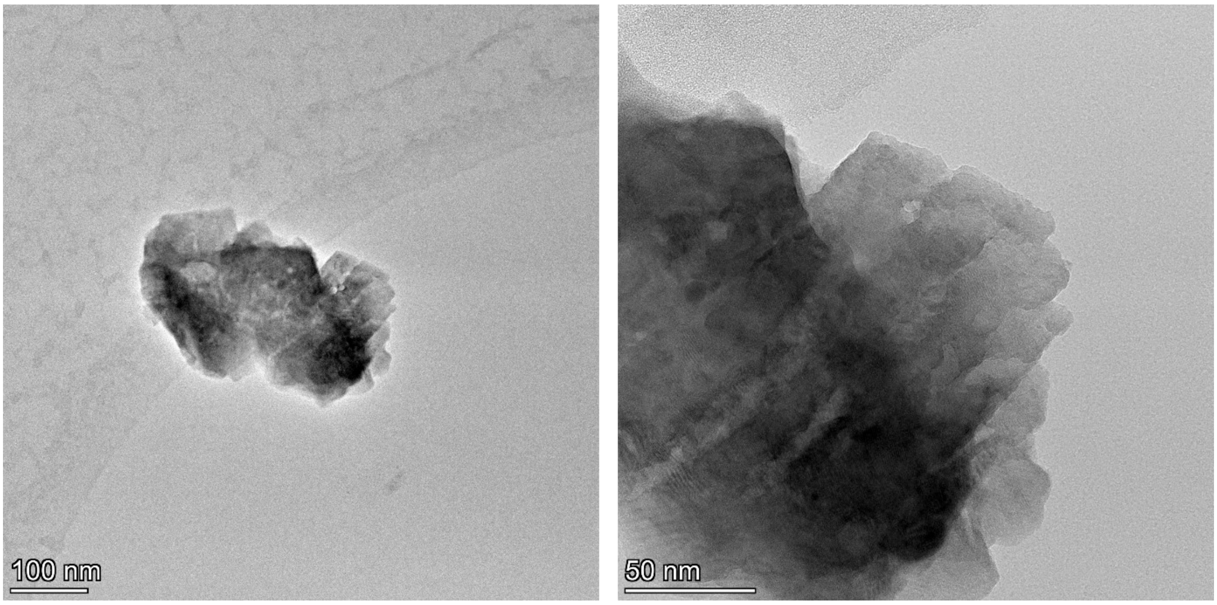

Figure S25. Bright-Field TEM (BFTEM) image of Cu-C catalyst.

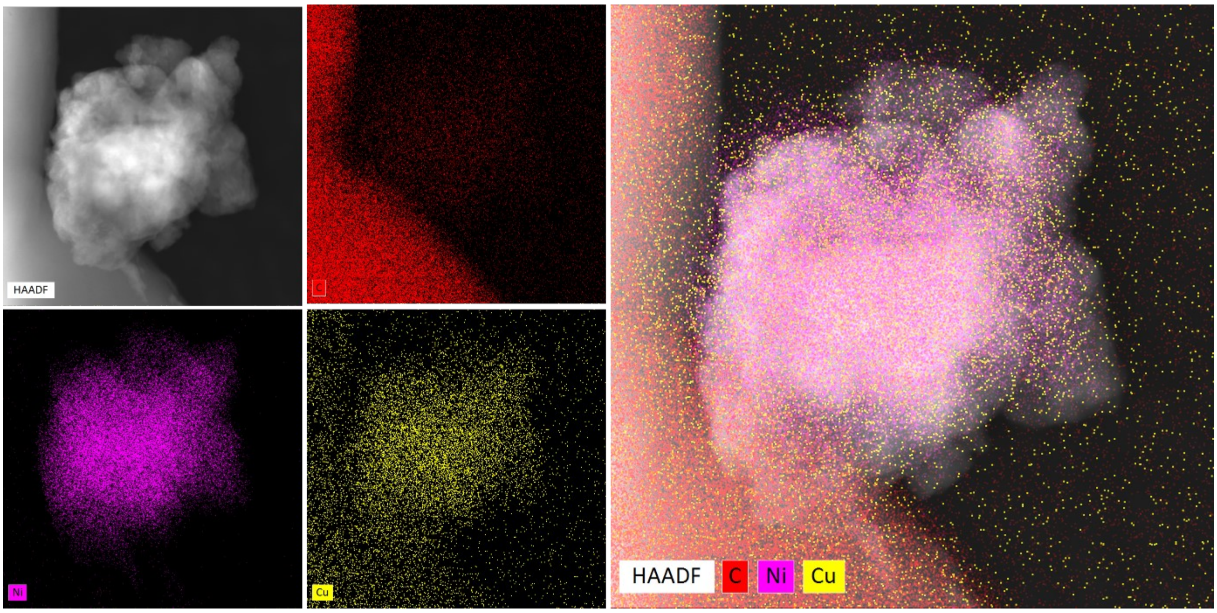

Figure S26. High-Angle Annular Dark-Field (HAADF-STEM) image of the bimetallic NiCu-C catalyst and corresponding STEM-EDX maps, confirming the uniform nanoscale intermixing of Ni and Cu.
